# Intrinsic functional connectivity delineates transmodal language functions

**DOI:** 10.1162/IMAG.a.25

**Published:** 2025-06-10

**Authors:** Joseph J. Salvo, Nathan L. Anderson, Rodrigo M. Braga

**Affiliations:** Ken and Ruth Davee Department of Neurology, Northwestern University Feinberg School of Medicine, Chicago, IL, United States; Department of Psychology, Northwestern University, Chicago, IL, United States

**Keywords:** functional connectivity, speech, language, auditory cortex, distributed association networks, resting state

## Abstract

Communication involves the translation of sensory information (e.g., heard words) into abstract concepts according to abstract rules (e.g., the meaning of those words). Accordingly, using language involves an interplay between*unimodal*brain areas that process sensory information and*transmodal*areas that respond to linguistic input regardless of the input modality (e.g., reading sentences or listening to speech). Previous work has shown that intrinsic functional connectivity (iFC), when performed within individuals, can delineate a distributed language network (LANG) that overlaps in detail with regions activated by a reading task. The network is widely distributed across multiple brain regions, recapitulating an organization that is characteristic of association cortex, and which suggests that the LANG network serves transmodal, not unimodal, functions. Here, we tested whether LANG encapsulates transmodal functions by assessing its degree of overlap with two language tasks: one auditory (i.e., listening to speech) and one visual (i.e., reading sentences). The results show that the LANG network aligns well with regions activated by both tasks, supporting a transmodal function. Further, the boundaries of the distributed language network along the lateral temporal cortex serve as a good proxy for the division between transmodal language and unimodal auditory functions: listening to sounds (i.e., filtered, incomprehensible speech) evoked activity that was largely outside of the LANG network but closely followed the network boundaries. These findings support that individualized iFC estimates can delineate the division between sensory-linked and abstract linguistic functions. We conclude that within-individual iFC may be viable for language mapping in individuals with aphasia who cannot perform language tasks in the scanner.

## 1 Introduction

Language allows us to communicate the same idea through multiple senses, including vision (reading), hearing (speech), or touch (braille). In each case, sensory percepts (i.e., words) are translated into their associated meaning according to linguistic rules. Thus, communication requires the coordination of a broad array of cognitive processes, including*unimodal*functions (i.e., those concerned with processing information from a given sense, such as visual, auditory, or tactile input) but also*transmodal*functions (i.e., those which are not exclusive to a particular sensory modality). In language processing, these transmodal functions may include syntactic and lexical processes that are likely shared across different forms of language input. Accordingly, brain mapping studies that use auditory or visual linguistic stimuli have often reported overlapping activity (e.g.,Binder et al., 1997;Fedorenko et al., 2010;Scott et al., 2017;Yuan et al., 2023). A recent proposal unites these shared regions as a “core language network” that is sensitive to linguistic stimuli regardless of input modality (Fedorenko et al., 2024; see alsoFedorenko & Thompson-Schill, 2014), but which interacts with modality-specific regions that provide sensory input and enable motor output (Lu et al., 2021). In this framework, the core language network sits in between unimodal sensory regions and widely dispersed association regions that encode the meaning of words (Huth et al., 2016).

Recent improvements in functional magnetic resonance imaging (fMRI) have allowed estimation of brain networks within individuals through repeated sampling (Braga & Buckner, 2017;Gordon et al., 2017;Laumann et al., 2015). The within-individual estimates allow better separation of networks which are blurred together by a group-wise approach that averages data across individuals (Braga & Buckner, 2017). We showed that within-individual intrinsic functional connectivity (iFC;Biswal et al., 1995) can delineate a distributed language network (LANG) from resting-state data, despite the absence of a language task (Braga et al., 2020; see alsoBranco et al., 2020;Glasser et al., 2016;Hacker et al., 2013;Lee et al., 2012;Tomasi & Volkow, 2012;Turken & Dronkers, 2011). The LANG network included prominent regions within the inferior frontal cortex, at or near the classic Broca’s area, and in the lateral temporal cortex, at or near the classic Wernicke’s area. In addition, we noted that the network also included regions in the supplementary motor area (SMA), the temporopolar region, and possibly ventral temporal cortex (Braga et al., 2020;Li et al., 2024;Nobre & McCarthy, 1995;Thomas et al., 2023). Confirming that this network serves linguistic functions, we showed that the full set of regions activated more strongly when participants read written sentences compared with lists of pronounceable, made-up words (i.e., pseudowords). This suggested that the whole distributed network is engaged during the processing of written sentences, and further supported that the network remains connected even in the absence of an overt language task.

An interesting observation was that when the full set of regions was considered, the organization of the LANG network recapitulated a parallel distributed network motif that is proposed to be characteristic of association cortex. Based on tracer studies of the macaque brain,Goldman-Rakic (1988)proposed that a parallel distributed network architecture, in which multiple association regions throughout frontal, parietal, temporal, and midline cortices are reciprocally interconnected, supports associative functions through brain-wide interactions. This architecture is fundamentally different from the stricter hierarchically connected networks that serve unimodal sensory and motor functions (Felleman & Van Essen, 1991;Mesulam, 1998). The distributed nature of LANG, therefore, suggests that the network serves transmodal, rather than unimodal, language functions.

Given the aforementioned findings that overlapping frontotemporal regions are activated for both auditory and visual language tasks (e.g.,Scott et al., 2017), we investigated the degree to which the LANG network, when defined using task-free resting-state data within individuals, overlapped with transmodal or unimodal language regions. Our previous study (Braga et al., 2020) used only visual stimuli (fromFedorenko et al., 2010) which aimed to isolate higher-level aspects of language related to lexical (i.e., linking sensory percepts with word-level meanings) and combinatorial processes (i.e., linking multiple words into phrase- and sentence-level meanings), while controlling for sensory processes (i.e., reading combinations of letter strings that did not have word- and sentence-level meaning). Interestingly, this visual language localizer activated lateral temporal regions that encircled primary auditory areas and Heschl’s gyrus, despite the absence of auditory input (Braga et al., 2020;Fedorenko et al., 2010). This implies that these lateral temporal LANG regions may serve transmodal language processes despite their proximity to auditory processing hierarchies (Rauschecker & Scott, 2009).

Here, we performed network estimation within individuals using iFC, then assessed whether the iFC-defined LANG network overlapped with activity evoked by visual (i.e., reading sentences;Fedorenko et al., 2010) and auditory language tasks (i.e., listening to speech;Scott et al., 2017) in those same individuals. We also assessed how the LANG network boundaries related to regions active during auditory processing of incomprehensible speech sounds.

## 2 Materials & Methods

### 2.1 Subjects and sessions

Ten adults (six female, ages 22–36 years, mean age 26.6 years, nine right handed) from the local community were recruited as part of the “Detailed Brain Network Organization” (DBNO) study. Details regarding this dataset have been previously described inKwon et al. (2025)andEdmonds et al. (2024). Participants had normal hearing and normal or corrected-to-normal vision, and no history of neurological illness. Informed consent was obtained from all participants for being included in the study. All participants provided written consent and were compensated for participation. Procedures were approved by Northwestern University’s Institutional Review Board. Participants were each invited to eight magnetic resonance imaging (MRI) sessions, each of which featured several cognitive tasks as well as a passive fixation (“resting-state”) task for network mapping using iFC. Prior to the first MRI session, participants were trained on all the tasks and were extensively coached about strategies for staying still in the scanner to improve data quality. While in the scanner, participants’ heads were padded using inflatable cushions to restrict head motion. Participants were also informed that the initial one to two MRI sessions would serve as a trial to assess compliance and whether they wanted to continue participation. Based on these criteria, two subjects were not invited back for additional sessions, leaving eight subjects who completed all eight MRI sessions (four female, ages 22–36 years, mean age 26.75 years, seven right handed). This led to a total of 60.8 hours of fMRI data collected (7.6 hours per participant), for precision functional mapping.

### 2.2 Task descriptions

#### 2.2.1 Passive fixation task (REST)

To allow estimation of large-scale networks within each individual using iFC, participants completed a passive fixation task in the scanner. Participants were shown a crosshair in the center of the screen and instructed to fixate on the crosshair for the duration of the task, keeping their eyes open and blinking normally. The task lasted ~7 minutes, and each participant completed 2 runs in each session, as the first and final runs of the session (total: 16 runs, ~112 minutes per participant). One participant (S2) completed an additional REST run to replace a poor-quality run that had been excluded in a prior session (seeSection 2.4).

#### 2.2.2 Reading-based language localizer (READLOC)

This task used visual stimuli (text) and lasted 5 minutes per run (fromFedorenko et al., 2010). In each trial, participants read a 12-word sentence or 12-pseudoword sequence on the screen, presented 1 word/pseudoword at a time. Participants were visually cued to press a button following each trial. Sentences and pseudoword sequences were presented in alternating blocks of 3 (1 block = 3 trials = 18.1 seconds), with 36 trials per run (18 sentences, 18 pseudoword). A crosshair was presented briefly between trials, as well as after every four blocks for a period comparable in length with the block duration (17.5 seconds). Participants were visually cued by a hand icon to press a button with their right index finger following each sequence, as a means of maintaining alertness. Note that the READLOC task contrast targeted reading of comprehensible sentences, while attempting to control for aspects such as eye movements, visual processing, attending to a screen, and pressing buttons. Importantly, the control condition may have preserved some suprasegmental aspects of reading, since the condition used pronounceable pseudowords as stimuli (e.g., if participants were sub-vocally articulating the pronounceable pseudowords).

#### 2.2.3 Speech-based language localizer (SPEECHLOC)

This task used auditory stimuli (speech) and lasted 7 minutes per run (based onScott et al., 2017). In each trial, participants listened to audio clips of normal intelligible speech and unintelligible, distorted speech. Audio clips were taken from recordings of TED talks, The Moth Radio Hour, and Librivox audiobooks. Two clips were taken from each recording, with one clip left intact and the second distorted by filtering as outlined below. This meant that the distorted clips preserved certain sound characteristics (speaker identity, intonation, speed, recording quality, etc.) to improve their effectiveness as control stimuli.

Each clip was edited to 18 seconds in duration, and was volume standardized and frequency equalized. All clips were compressed using a threshold of -40dB, and were transformed such that their root-mean-square equaled 0.0628. These values were chosen based on qualitative assessments of sound quality and volume by experimenter J.S. Stereo clips were converted to mono (same output for both ears). Clips were equalized using earphone-specific filters provided by the earphone manufacturer, Sensimetrics (Gloucester, USA). Without this filtering, clips sounded unnaturally shifted toward higher frequencies (“tinny”). We ultimately used the left-ear filter for both channels after pilot participants reported improved sound clarity.

For the distorted speech condition, half of the clips were filtered and combined with white noise to remove comprehensible speech, followingScott et al. (2017). To prevent filtering artifacts, dummy vectors 1/10th the length of each sound clip, with a magnitude matching the clip’s mean dB, were added to the start and end of each clip. Clips were lowpass filtered using a Butterworth infinite impulse response filter (IIR: Butterworth), with a pass frequency (Fpass) of 500 dB, a stop frequency (Fstop) of 600 dB, a passband ripple (Apass) of 1 dB, and a stopband attenuation (Astop) of 146 dB. White noise was generated by shuffling time points in the original clip and saving the resulting noise to a new vector. This vector was then transformed according to the amplitude envelope of the original clip, such that the volume of the noise was modulated by the volume of the voice. The noise vector was also lowpass filtered (IIR: Butterworth, Fpass = 7,500 dB, Fstop = 10,700 dB, Apass = 1 dB, Astop = 40 dB). Noise and voice were combined with a volume ratio of 1:2. The combined clip was lowpass filtered once more, using a threshold of 8,000 dB, to remove noise-related spikes. Finally, the clip was trimmed back to a standard duration of 18 seconds. To avoid clicking artifacts at the start and end of each sample, both intact and distorted clip volumes were ramped according to a y = x or y = 1-x slope, applied, respectively, to the clip’s first and last 0.01 seconds.

Importantly, although the distorted clips were incomprehensible (i.e., participants could not make out the actual words being spoken), the clips preserved certain properties of speech, such as prosody and intonation. Therefore, the SPEECHLOC task contrast targeted listening to comprehensible speech, while trying to control for other aspects of the task, such as attending to sounds, auditory processing of sounds with similar spectral content, pressing buttons. However, because the control condition included filtered, incomprehensible speech, it is possible that listening to other forms of incomprehensible speech, such as foreign languages, might activate different regions to those revealed by the SPEECHLOC contrast.

Audio was presented through Sensimetrics S14 MRI-compatible earphones. To ensure that the clips were audible over the scanner noise, at the start of each MRI session, prior to data collection, we tested sound volume while running a dummy version of the MR imaging sequence to mimic the conditions of the task. Participants were played two speech clips (one clear, one distorted) followed by the tone used as a button-press cue. Participants used the provided button box to adjust the volume to a comfortable level at which the speech could be clearly heard, and were asked to describe the topic of the clearly presented story.

Audio clips lasted 18 seconds and were followed by a tone lasting 0.15 seconds. Participants then had 2 seconds to press a button with their right index finger to register attention. Each task run included 8 clear and 8 distorted 18 seconds audio clips. Four clips were presented as a block (two clear, two distorted), with four blocks per run. A fixation cross (“+”) was presented between blocks for 14 seconds. Within each block, clips were presented in a counterbalanced order, for example, CDDC+DCDC+CDCD+DCCD (C: clear clip, D: distorted clip, +: fixation), ensuring an even distribution of conditions across the run. Block order was also counterbalanced across runs.

Participants completed additional tasks in each session that are not described in this report (seeEdmonds et al., 2024;Kwon et al., 2025).

### 2.3 MRI data acquisition

Data were collected on a Siemens 3.0 T Prisma scanner (Siemens, Erlangen, Germany) at the Center for Translational Imaging at Northwestern University’s Feinberg School of Medicine in Chicago. Two anatomical images were collected: a T1-weighted image in the first MRI session (TR = 2,100 ms, TE = 2.9 ms, FOV = 256 mm, flip angle = 8°, slice thickness = 1 mm, 176 sagittal slices parallel to the AC-PC line) and a T2-weighted scan in the second session (TR = 3,000 ms, TE = 565 ms, FOV = 224 mm x 256 mm, flip angle = 120°, slice thickness = 1 mm). Both scans included volumetric navigators (Tisdall et al., 2012) from the Adolescent Brain Cognitive Development study (Hagler et al., 2019). Functional MRI data were collected using a 64-channel head coil using a multi-band, multi-echo sequence with the following parameters: TR = 1,355 ms, TE = 12.80 ms, 32.39 ms, 51.98 ms, 71.57 ms, 91.16 ms, flip angle = 64°, voxel size = 2.4 mm, FOV = 216 mm x 216 mm, slice thickness = 2.4 mm, multiband slice acceleration factor = 6 (seeLynch et al., 2020;Poser et al., 2006).

### 2.4 Quality control

Head motion was estimated using FSL’s*MCFLIRT*(Jenkinson et al., 2002). Runs were automatically excluded from analysis if motion exceeded predetermined thresholds of framewise displacement (FD) > 0.4 mm or absolute displacement (AD) > 2.0 mm. Runs were flagged for visual inspection if motion exceeded more stringent thresholds (FD > 0.2 mm, AD > 1.0 mm), and the whole run was then excluded if motion could be clearly seen in the raw data. Runs for the two localizer tasks could also be excluded due to poor behavioral performance. For READLOC, runs with more than 5% missing button presses were excluded. For SPEECHLOC, runs with more than 20% missing button presses were excluded (as there were fewer total trials).

After quality control, each participant retained at least 10 REST runs (range: 10–16). For all participants except one (S5), half of the data were set aside for replication analyses (not used in this study), leaving 49–60 minutes of data for each participant (S1: 56 minutes; S2: 56; S3: 56; S4: 56; S5: 60; S6: 49; S7: 49; S8: 49).

For SPEECHLOC, at least seven runs were retained per subject (S1: 8; S2: 8; S3: 8; S4: 7; S5: 7; S6: 8; S7: 7; S8: 8) for a range of 49 to 56 minutes of data per participant. For READLOC, at least four runs were retained per subject (S1: 8; S2: 8; S3: 8; S4: 4; S5: 7; S6: 8; S7: 5; S8: 7) for a range of 20 to 40 minutes of data per participant.

### 2.5 MRI data preprocessing

Blood-oxygenation-level-dependent (BOLD) data were preprocessed using a custom processing pipeline “iProc” (Braga et al., 2019) optimized for within-individual alignment of data from multiple runs and sessions. Interpolation steps were combined in an effort to minimize blurring. Additional steps were included to account for the multi-echo data (outlined below). Each individual’s data were processed separately. The first 9 volumes (~12 seconds) of each run were discarded to remove a T1 attenuation artifact. Next, a mean BOLD template was created as an interim stage for data registration by averaging the first echo of all included runs of all tasks, to reduce bias toward any one particular run. The participant’s T1 anatomical image was used to create a native space template. These templates were used to build four matrix transforms to align each BOLD volume (1) to the middle volume of the same run for motion correction, (2) to the mean BOLD template for cross-run alignment, (3) to the native space template, and (4) to the 152 1-mm atlas (Mazziotta et al., 1995) from the Montreal Neurological Institute (MNI) International Consortium for Brain Mapping (ICBM). The four transforms were composed into two matrices, which were then applied to the original volumes to register all volumes to the T1 anatomical template (matrices 1-3) and to the MNI atlas (matrices 1–4) in a single step. The MNI registration was used in the nuisance regression steps applied to the resting-state runs (*see *Section 2.6below). Visual checks were incorporated into each registration step of the pipeline.

Motion correction transforms were calculated using rigid-body transformation based on the first echo, which had less signal dropout and better preserved the shape of the brain. Registration matrices were calculated based on the first echo, and then applied to all the echoes. Once data were projected to the T1 native space, the five echoes were combined to approximate local T2* by weighting each echo according to its temporal signal-to-noise (tSNR) ratio and echo time (Heunis et al., 2021). Briefly, the tSNR was calculated for each echo, which was then weighted by the echo time. This weighted tSNR was then divided by the sum of all weighted tSNRs (i.e., for all echoes). The resulting image was multiplied by the echo’s original intensity image, and these were summed across all echoes to create the final optimally combined image.

### 2.6 Functional connectivity preprocessing

Nuisance variables were calculated for each run, by extracting signal averages from deep white matter and from cerebrospinal fluid using masks hand drawn in MNI space that were back projected to the native space. Additional nuisance variables included six motion parameters, whole-brain (global) signal, and their respective temporal derivatives. Data were then bandpass filtered using a range of 0.01–0.1 Hz using*3dBandpass*(AFNI v2016.09.04.1341;Cox, 1996;^2012^). Next, data were projected to a standard cortical surface (fsaverage6, 40,962 vertices per hemisphere;Fischl et al., 1999), and smoothed with a 2 mm full-width at half-maximum kernel along the surface. This kernel size was chosen based on prior work (Braga & Buckner, 2017;Braga et al., 2019) to retain functional anatomic detail while limiting noise (i.e., speckling) in the functional connectivity maps.

### 2.7 Network estimation

Prior to any analysis of the task data, the independent REST data were used to create subject-specific estimates of large-scale networks using iFC following our previously used procedures (Braga & Buckner, 2017;Braga et al., 2020). First, functional connectivity matrices were calculated for each REST run by computing vertex–vertex Pearson’s product–moment correlations. The matrices were then z-normalized using the Fisher transform, averaged across all runs within each individual, then converted back to r values using the inverse Fisher transform. These within-individual average matrices were used to perform a seed-based analysis. Seeds were manually selected in the left hemisphere to delineate eight networks, including LANG (seeBraga & Buckner, 2017;Braga et al., 2020;Edmonds et al., 2024;Kwon et al., 2025), and in the right hemisphere to identify the auditory network, AUD (to ensure the left-hemisphere correlated regions observed were not due to local autocorrelation effects). Left-hemisphere language seeds yielded comparable correlation patterns whether chosen generally in lateral temporal cortex vs. specifically in posterior lateral temporal cortex, closer to the typical site of Wernicke’s area (Tremblay & Dick, 2016). We then used a multi-session hierarchical Bayesian Model (MS-HBM) approach to define networks using a data-driven algorithm applied to the same data (Kong et al., 2019). This method stabilizes individual-specific network estimates by integrating priors from multiple levels (e.g., group-level, cross-individual, and cross-run variation). For each individual, we generated clustering solutions with k values (i.e., number of clusters) between 12 and 18, and selected the lowest level of clustering that separated the networks of interest as defined by the seed-based approach. The MS-HBM algorithm also allowed definition of networks beyond the*a priori*selected networks, including those covering auditory cortex (surrounding bilateral Heschl’s gyrus; AUD) and ventral somatomotor cortex (surrounding bilateral inferior central sulcus).

### 2.8 Task activation maps

Data from the localizer tasks were analyzed using FSL’s*FEAT*(Woolrich et al., 2001). Each hemisphere of each surface-projected task run was input into a separate general linear model. The task was modeled using explanatory variables that were convolved with a double-gamma hemodynamic response function using*FEAT*.

For READLOC, regressors were specified that included all 18.1-second blocks of reading for each of the 2 block types (real words and pseudowords). Beta values were calculated for the real words and pseudowords conditions, and a contrast of parameter estimates was calculated between these conditions for each run. The contrast maps were normalized into z values by FEAT, and then averaged across runs for a given task and individual.

For SPEECHLOC, regressors were specified that included all 18-second presented audio clips for each of the 2 speech types (comprehensible and distorted). Beta values were calculated for the comprehensible and distorted speech conditions, and a contrast of parameter estimates was calculated between the conditions for each run. The contrast maps from each run were normalized into z values by*FEAT*, and then averaged across all runs for a given task and individual.

An additional contrast map, AUDLOC, focused on the distorted speech condition of SPEECHLOC, contrasted implicitly against the fixation baseline periods. This contrast was used to localize auditory cortex (i.e., regions active during sound presentation compared with baseline). Because the distorted speech condition preserved some features of speech sounds, such as prosody and intonation, this contrast also likely activated speech-selective regions sensitive to these features that may lie outside primary and secondary auditory cortex (e.g.,Norman-Haignere et al., 2015).

Task activity was initially visualized using qualitatively chosen thresholds. Threshold values were chosen for each participant to highlight larger regions containing higher values while minimizing smaller regions and speckling (indicating noise) containing lower values. Based on this, thresholds were picked individually for each subject and task contrast map, prior to any assessment of overlap. We started at a threshold of z > 2.5, and lowered the threshold if the activation map was smaller than expected (e.g., comparing withBraga et al., 2020). For the AUDLOC task map, we started at a higher threshold of z > 3.0 and used this for all subjects. To ensure that the results were not specific to these particular thresholds, we also created maps thresholded at the 95th percentile for each participant (Supplementary Figs. S1–S3), which produced very similar maps. Given their similarity, we used the qualitatively chosen thresholds for all further analyses.

To ease visualization of overlap, we binarized the thresholded maps from each of the task contrasts. First, we overlaid the maps from the two active language localizers (READLOC, SPEECHLOC) to identify regions responding to linguistic stimuli from both modalities. Second, we overlaid the maps from the READLOC and AUDLOC contrasts to test the degree of overlap between transmodal language and auditory regions with the iFC-estimated LANG network. The latter two contrasts were chosen so that the estimate of transmodal language regions (from READLOC) was from independent data to the maps of auditory regions (from AUDLOC), and to ensure that any overlap or lack thereof was not due to the contrasted conditions. Third, we overlaid maps from READLOC, SPEECHLOC, and AUDLOC altogether to visualize the alignment between all tested conditions with the language and auditory networks (Fig. 6).

### 2.9 Assessing overlap

We quantified how closely vertices that overlapped between contrasts (READLOC and SPEECHLOC, READLOC and AUDLOC) aligned with the LANG network in the left hemisphere. For each candidate vertex, we first determined whether the vertex fell within the boundaries of the LANG network (distance of 0). If not, all neighboring vertices on the surface mesh were identified (those sharing a face with the candidate vertex). If any of these vertices fell within the LANG network, the distance for the candidate vertex was 1. If not, we identified all vertices sharing a face with these neighboring vertices (second-level neighbors), and so on. This process was repeated until a vertex within the LANG network was located, and a distance value was assigned to the candidate vertex. In essence, this determined the shortest distance between each candidate vertex and the LANG network.

Additionally, we tested whether alignment between activity and a given network was greater than chance by implementing a spin permutation testing approach (Alexander-Bloch et al., 2018). For each comparison, we first calculated the “observed” Dice overlap coefficient between vertices active during the task contrast of interest (SPEECHLOC, LANGLOC, AUDLOC) and the networks defined using functional connectivity (LANG, AUD). Next, we rotated the network estimate around the cortical surface by a random amount 50,000 times and calculated the resulting “permuted” Dice coefficient of each iteration. This allowed us to compare the observed overlap values with the permuted distribution to assess whether the observed values were greater than expected due to chance.

## 3 Results

### 3.1 The distributed language network was defined using multiple intrinsic functional connectivity approaches

**Fig. 1.**
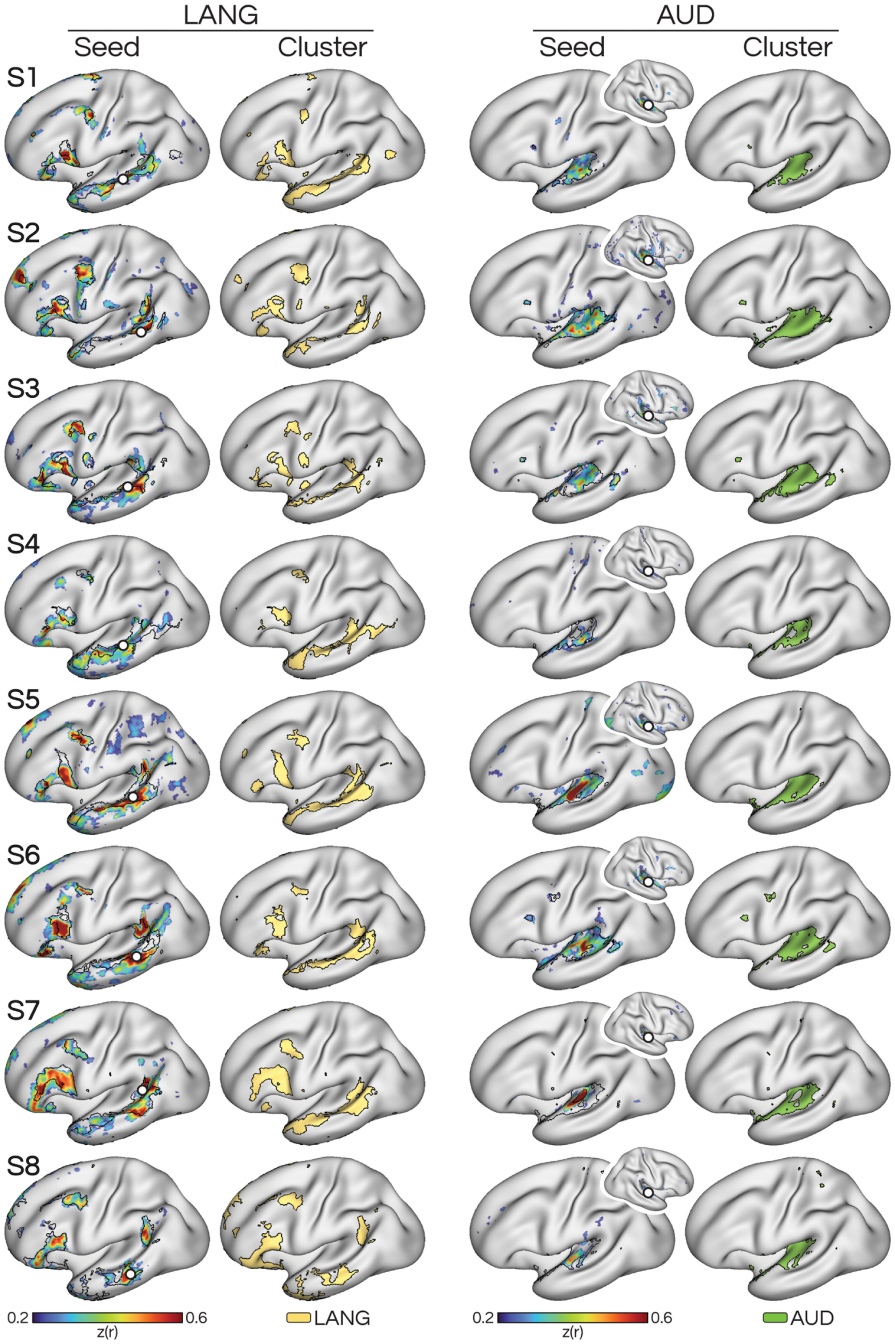
Within-individual functional connectivity estimates reveal separate, adjacent networks in superior temporal cortex. (Left) Seeding in left lateral temporal cortex revealed a correlated network that includes regions classically associated with language processing (LANG; seeBraga et al., 2020), including in inferior frontal cortex. The same network was also revealed by a data-driven clustering analysis, which provided converging estimates of the network (see how black boundaries of the clustering solution overlap with regions showing high correlation in the seed-based map). (Right) Seeding in a nearby analogous location in right lateral temporal cortex revealed a different network (AUD) that includes left hemisphere regions associated with auditory processing. This network was also confirmed as a distinct network by the clustering analysis. The two networks contain adjacent regions throughout the superior temporal cortex in the left hemisphere (see zoom-ins inFig. 4).

We defined large-scale networks*a priori*in each individual using iFC analysis of the independent passive fixation (i.e., “resting-state”) data. Seeds were chosen in lateral temporal cortex in the left hemisphere to identify the canonical left-lateralized language network (LANG). Seeds were also chosen in the right lateral temporal cortex to identify the primary auditory network (AUD) in the contralateral hemisphere (Fig. 1). Note that left-hemisphere seeds were also able to define the AUD network, despite being adjacent to the seed used to define LANG (not shown). As a complementary means to define networks, correlated vertices were clustered together using a data-driven approach (Fig. 1), with the number of clusters selected based on visual correspondence with the seed-based iFC maps, respecting that networks may be over split or over lumped at different clustering levels. Candidate LANG and AUD networks were identified at a clustering solution of 14 clusters for subjects S1–S7. For S8, the candidate LANG network from the*k*= 14 solution was adjacent to but did not align well with the seed-based maps of the LANG network nor with the language-localizer maps. Therefore, for this participant, we*post hoc*changed the LANG and AUD estimates, which were instead taken from the 15-cluster solution. Both analysis methods revealed a network comprising classic language regions, including in inferior frontal, lateral temporal, and posterior middle frontal cortices. Further, the results distinguished the LANG network from a neighboring network in lateral temporal cortex, AUD, that likely covers primary and secondary auditory areas.

### 3.2 Confirmation that the distributed language network is activated by a visual (reading-based) language task

**Fig. 2.**
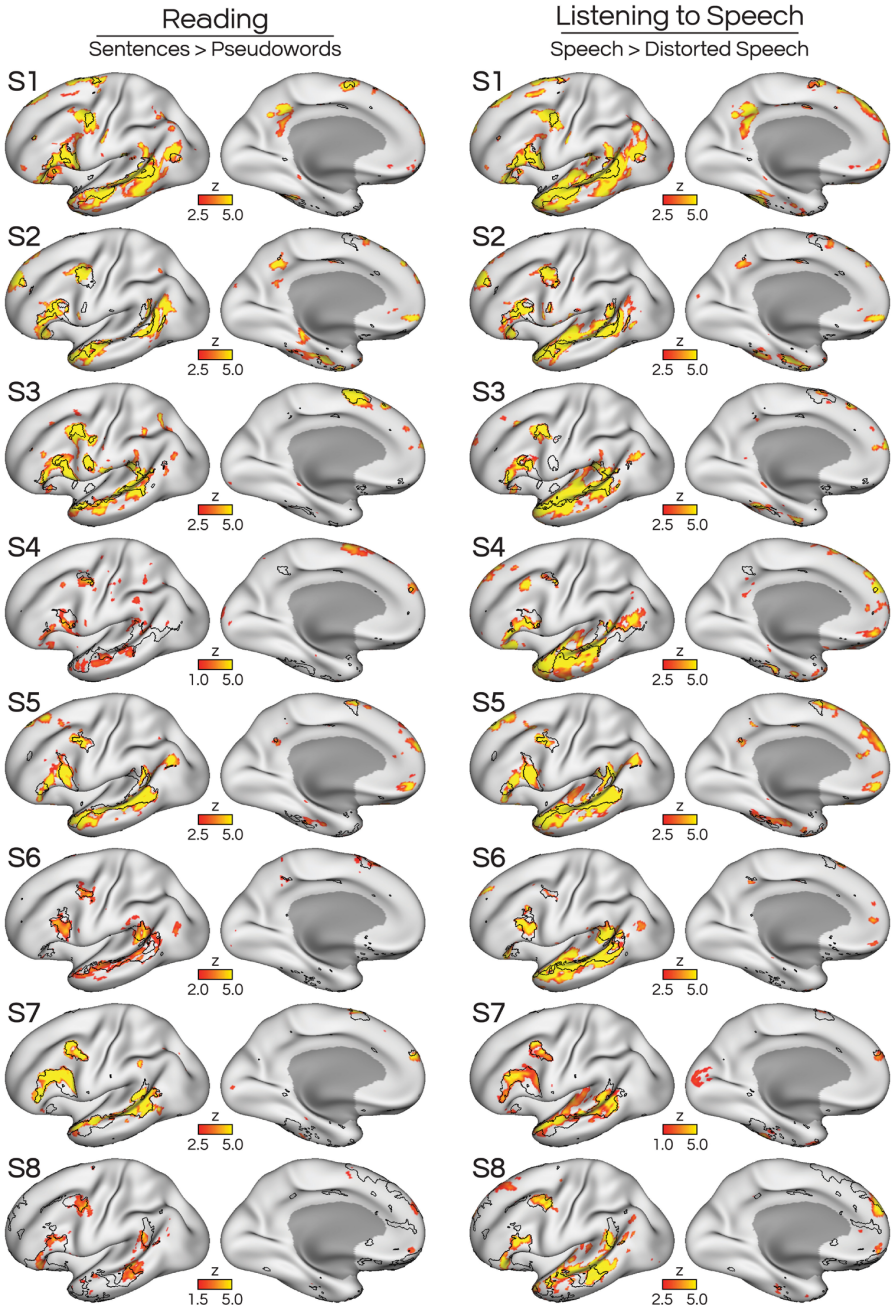
Visual language input (reading) and auditory language input (listening to speech) both robustly activated the distributed LANG network. (Left) We replicated the results ofBraga et al. (2020)in a new dataset, by showing that a reading task (READLOC; reading sentences contrasted against reading pseudoword sequences) elicited activity that overlapped with the iFC-derived estimate of LANG (see black borders from clustering solutions shown inFig. 1). Detailed correspondence between iFC- and task-based estimates was seen in lateral temporal, inferior frontal, and posterior middle frontal cortex, as well as the pre-supplementary motor area (SMA). Note that for two subjects (S4, S8), the READLOC task did not elicit strong activation maps, so thresholds were lowered for these subjects to reveal a similar distribution of activated regions. (Right) An auditory version of the language localizer task (SPEECHLOC; contrasting listening to sentences against listening to distorted speech) revealed a similar distribution of regions, which also overlapped with the iFC-defined LANG network in multiple cortical locations. The SPEECHLOC map also included broader lateral temporal activity which likely extended into auditory regions, as explored in subsequent figures. InSupplementary Figures S1 and S2, we show the same maps using a 95^th^percentile threshold for all participants.

We first replicated the results ofBraga et al. (2020)by showing that the iFC-defined LANG network we selected supports linguistic functions. Analysis of the READLOC task showed greater activity within the LANG network while participants read comprehensible sentences, contrasted with reading sequences of pseudowords (Fig. 2, left). Notably, regions showing the greatest activity fell within the bounds of the LANG network, and in multiple regions, the boundaries of the LANG network overlapped closely with activity evoked by the task. These results replicated that the LANG network is engaged at the network level during a reading task.

### 3.3 The distributed language network encompasses transmodal language functions

To establish whether the iFC-defined LANG network serves a transmodal language function, we analyzed the SPEECHLOC task data, which contrasted listening to clear, comprehensible speech with listening to distorted, incomprehensible speech. Increased activity was observed for this contrast throughout the LANG network, including in the lateral superior temporal cortex, inferior frontal cortex, and posterior middle frontal cortex (Fig. 2, right). Some subjects (S1, S2, S3, S5) also showed activity in the SMA and anterior superior frontal cortex, typically visible on the medial surface. Active regions consistently fell within the boundaries of the iFC-defined LANG network, often closely matching each subject’s unique functional anatomy (i.e., location and shape of network regions). The location and extent of these regions were similar whether using qualitative or quantitatively defined thresholds (Supplementary Figs. S1–S3).

**Fig. 3.**
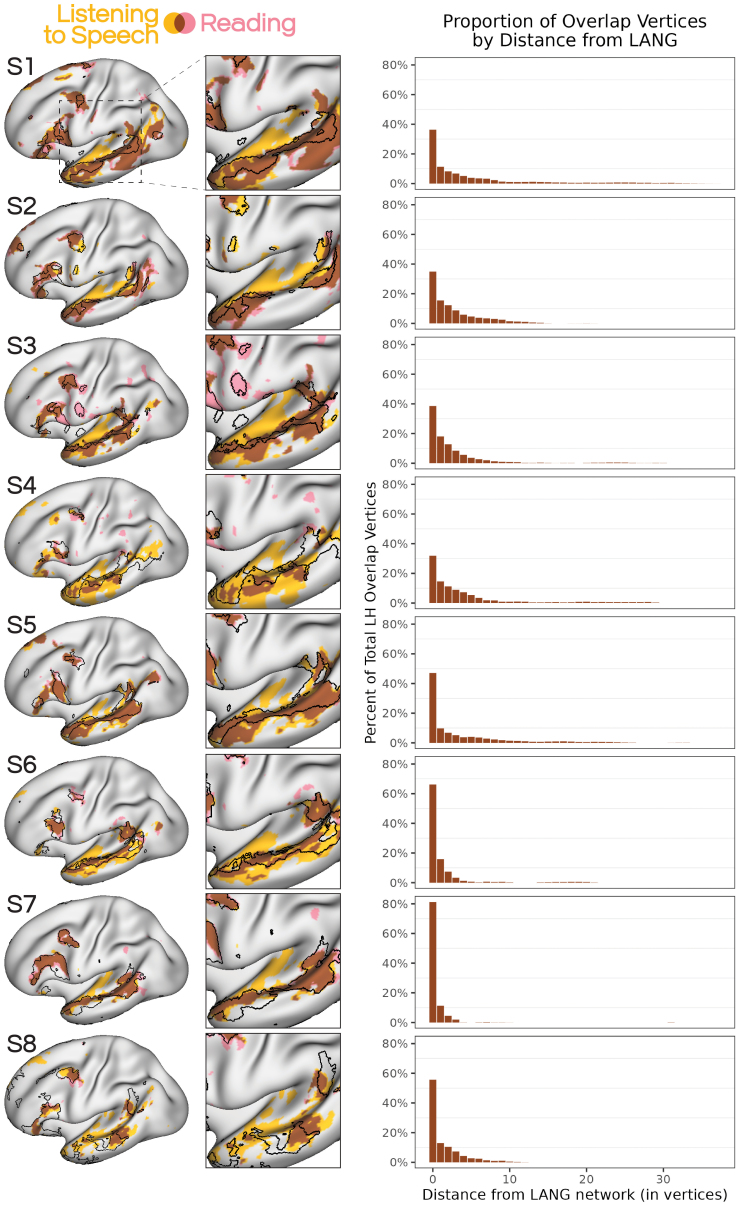
Overlap between activity elicited by reading and listening to speech was primarily within the distributed LANG network. (Left) Regions activated by the auditory SPEECHLOC task overlapped extensively with those activated by the visual READLOC task. This overlap implies that the LANG network encapsulates transmodal language functions that engage with linguistic stimuli regardless of input modality. (Right) Vertices showing overlap between the two task maps were quantified with respect to their proximity to the LANG network; in all subjects, the majority of overlap vertices were within the LANG network (a distance of 0) and the remainder were within a short distance of LANG, with the incidence of overlap vertices falling off with greater distance from the network.

We next created an overlap map between activation during reading- (READLOC) and speech-based comprehension (SPEECHLOC) in each subject. Despite the language input being from two different modalities, we observed extensive overlap of these two tasks, replicatingFedorenko et al. (2010)andScott et al. (2017). However, here we also observed that this overlap between the tasks occurred mostly within LANG network regions (Fig. 3, left). To quantify this alignment, we took the surface vertices that showed overlap between the SPEECHLOC and READLOC contrasts, and calculated their distance to (i.e., number of vertices away from) the LANG network. Across all eight subjects, the majority of vertices fell within the LANG network, and those outside were a short distance away (Fig. 3, right). Spin-testing revealed that in all participants the degree of alignment between LANG and the vertices active for both language tasks was greater than seen in at least 49,943 alternate rotations of LANG, much greater than expected by chance (Supplementary Fig. S4), and falling on the high end of the data distribution. By comparison, the alignment between AUD and the task-overlap vertices was toward the center of the distribution. This consistency confirmed that the LANG network likely supports transmodal language processes, and likely encapsulates regions activated by linguistic input regardless of modality.

For the SPEECHLOC contrast, we also saw activation that extended into the Sylvian fissure that was not seen in READLOC, suggesting that the SPEECHLOC contrast was also revealing activity within unimodal auditory regions as captured by the AUD network (Supplementary Fig. S5). These observations raised the prospect that the boundaries of the iFC-defined LANG network might provide a relatively accurate delineation between modality-specific (i.e., auditory) and transmodal aspects of language processing.

### 3.4 Activity while listening to incomprehensible speech activates primary auditory cortex

**Fig. 4.**
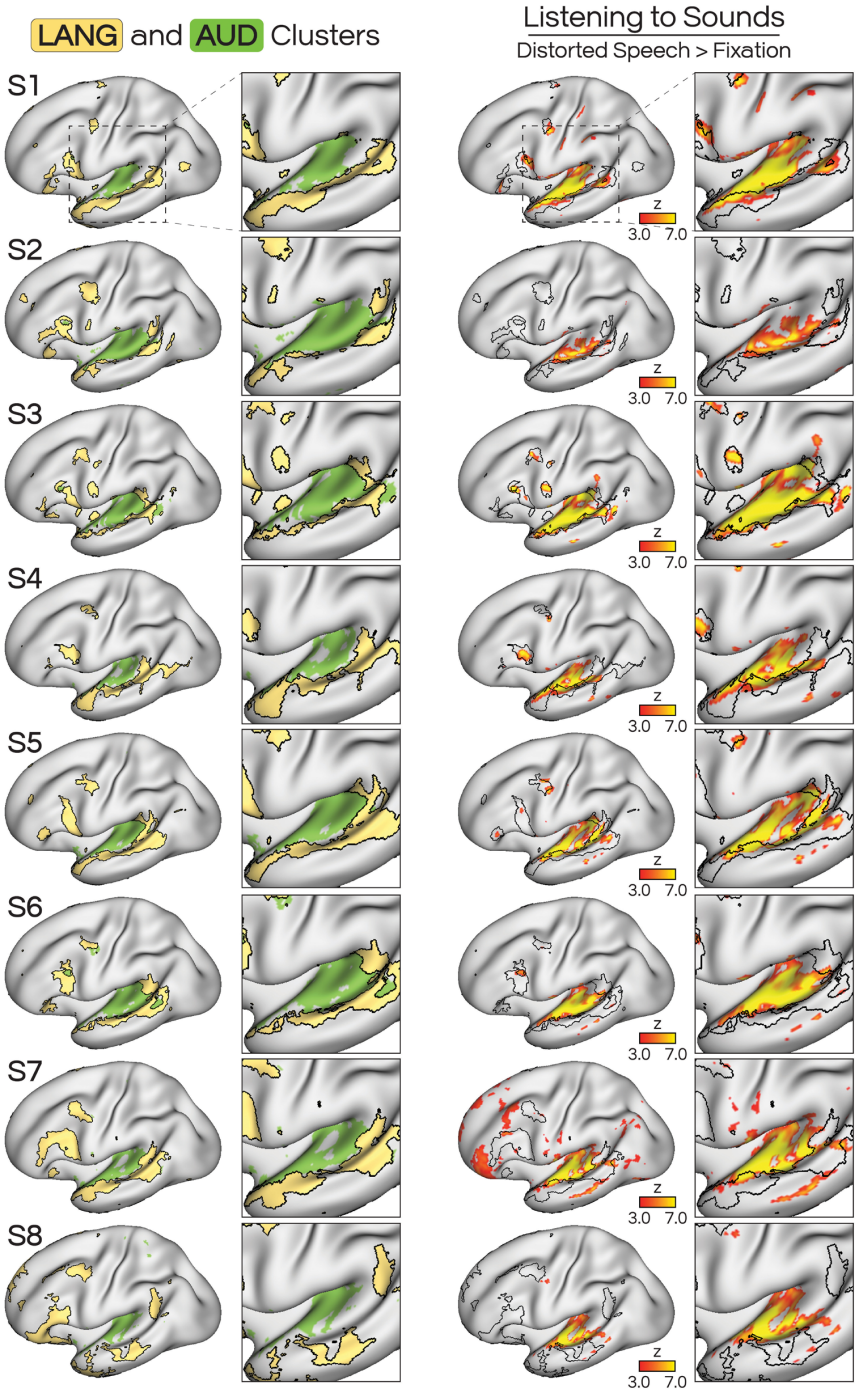
Auditory non-linguistic input (listening to distorted, incomprehensible speech) activated the AUD network, with limited activation of the adjacent LANG network. (Left) The iFC-defined AUD and LANG networks included adjacent regions, with LANG (yellow) encircling AUD (green) throughout the dorsal bank of the lateral temporal cortex. (Right) The AUDLOC contrast contrasted listening to distorted speech against a passive fixation baseline as a localizer for auditory cortex. This map showed that listening to sounds revealed activity mainly within the AUD network, partly extending into the neighboring LANG network to varying degrees across subjects. We explored this overlap further inFigure 5.

As a functional localizer for unimodal auditory regions, in the AUDLOC contrast we contrasted the distorted speech condition from the SPEECHLOC task with the fixation baseline of the same task. The resulting map revealed bilateral activation of superior lateral temporal areas that were at or near Heschl’s gyrus (Fig. 4), indicating that this was an effective contrast for isolating auditory cortex. Additional smaller regions of activity were also found in prefrontal cortex in some subjects (discussed later). Listening to distorted speech most extensively recruited regions in the AUD network, outside of the LANG network (Supplementary Fig. S6). Spin-testing revealed that in all participants, the degree of alignment between AUD and the vertices active for AUDLOC was greater than seen in at least 49,807 alternate rotations of AUD (Supplementary Fig. S7). The alignment between LANG and AUDLOC-active vertices was also highest for the observed orientation, however, the overlap was less between AUDLOC/LANG than for AUDLOC/AUD. We did observe some degree of overlap into the LANG network, to a varying amount across subjects (explored later).

### 3.5 The boundaries of the distributed language network separate unimodal and transmodal functions

**Fig. 5.**
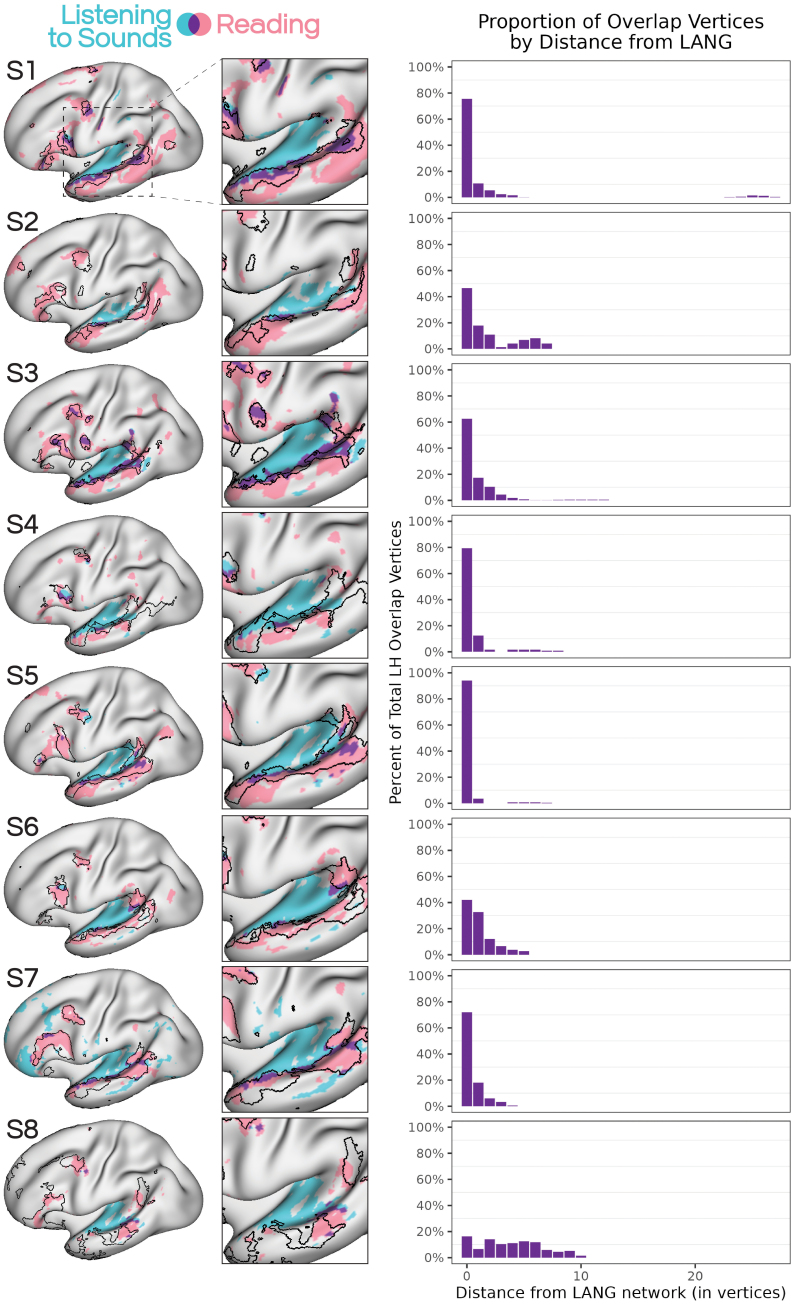
The boundaries of the LANG network in superior temporal cortex delineated between auditory and transmodal functions. (Left) Regions that showed increased activity during reading sentences (from READLOC; seeFig. 2), chosen here to represent transmodal language functions (seeFig. 3), were overlaid with regions activated by listening to non-linguistic sounds (the AUDLOC contrast; seeFig. 4). The insets show that the borders of LANG in many cases captured the transition between unimodal auditory and transmodal language functions. In other cases, overlap was sometimes seen between the two maps, however, this overlap was mostly within the bounds of the LANG network. This overlap could be due to technical reasons (e.g., blurring) or the fact that the distorted speech stimuli preserved some features of speech, such as the intonation and prosody of the speaker, even though the spoken words were made incomprehensible. (Right) Vertices showing overlap between the two maps were quantified with respect to their proximity to the LANG network; in subjects 1–7, the majority of overlap vertices were within the LANG network (a distance of 0), and the others were few vertices away.

We explored the extent to which the iFC-defined LANG network separated transmodal and unimodal functions. To do so, we compared the thresholded, binarized maps from the READLOC contrast with the AUDLOC contrast (Fig. 5, left). First, we observed that the boundaries of the LANG network provided a remarkably good proxy for the demarcation of auditory non-linguistic from transmodal linguistic functions. In many cases, the areas of overlap between the maps were few, and the adjacent regions from each task were positioned close to, if not exactly at, the boundaries of the LANG network (see insets inFig. 5). In some cases, the two maps overlapped, indicating that some regions that responded to the distorted speech stimuli were also recruited by the reading task. Notably, the regions that showed overlap between these maps were restricted almost entirely to the LANG network. To quantify this, we took the surface vertices that showed overlap between READLOC and AUDLOC, and calculated their distance to (i.e., number of vertices away from) the LANG network. Across seven of the eight subjects, the majority of the vertices fell within the LANG network, and those outside were a short distance away (Fig. 5, right). For S8, most vertices fell just outside the LANG network, possibly related to issues with network definition in this subject (seeSection 2.7).

Spin-testing revealed that in all participants, the degree of alignment between LANG and the vertices active for both READLOC and AUDLOC was greater than seen in at least 42,769 alternate rotations of LANG (Supplementary Fig. S8). By comparison, the alignment between AUD and these vertices was toward the center of the distribution.

**Fig. 6.**
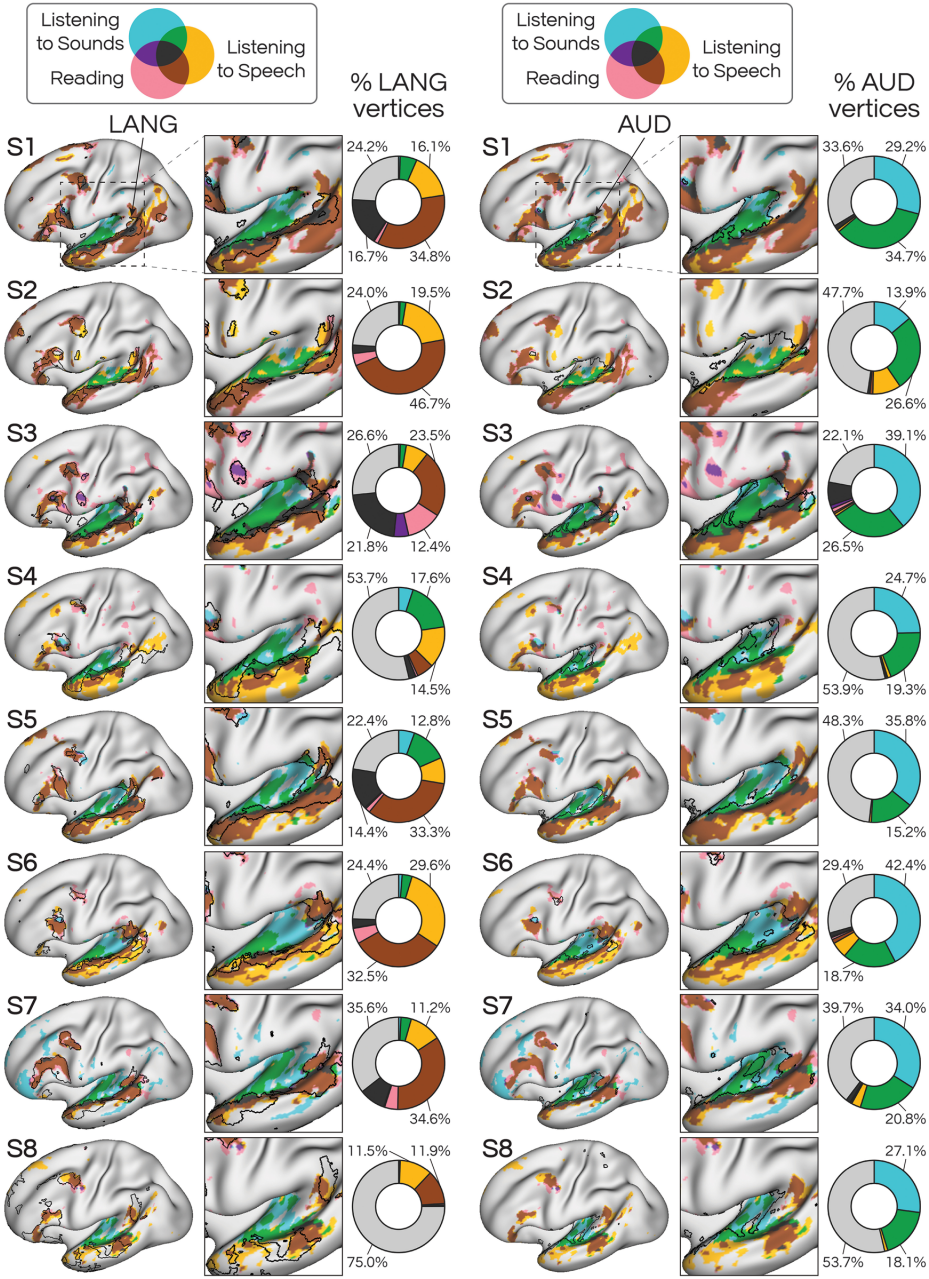
Overlay of activity from all three task contrasts (AUDLOC, READLOC, SPEECHLOC) with borders of the language network (LANG; left) or auditory network (AUD; right). Pie charts indicate percentages of the network active for each condition. Generally, activity observed within LANG was most related to SPEECHLOC (listening to comprehensible speech) or both SPEECHLOC and READLOC (reading comprehensible sentences; yellow, brown), while activity within AUD was most related to AUDLOC (listening to incomprehensible speech) or both AUDLOC and SPEECHLOC (blue, green). This distinction supports the functional separation between the LANG and AUD networks, with LANG associated with transmodal language processes and AUD associated with auditory perception. Qualitative thresholds were used as shown inFigures 2–4.

These results revealed that iFC provides a remarkably accurate delineation between unimodal auditory and transmodal language functions along the left lateral temporal cortex. To further visualize this relationship, we mapped activity from each of the three conditions (READLOC, SPEECHLOC, AUDLOC) on the same surface, overlaid with the borders of LANG and AUD (Fig. 6). We observed that LANG primarily included activity from the language tasks (READLOC, SPEECHLOC; see red, yellow, and brown regions), while AUD primarily included activity during tasks that used auditory stimuli (SPEECHLOC, AUDLOC; see yellow, blue, and green regions). As noted withFigure 5, a portion of LANG was activated by the AUDLOC contrast (see blue, green, purple and black inFig. 6, left). One possible explanation for this is that the AUDLOC active condition, which included listening to filtered speech, preserved suprasegmental aspects of speech (e.g., intonation, prosody) and, therefore, activated speech-sensitive lateral temporal regions. It is worth noting that much of this area was also active during SPEECHLOC, which used the filtered speech as a baseline. Thus, this area would have displayed greater activity during comprehensible speech than during the incomprehensible speech (green and black regions). This is compatible with these regions being speech sensitive, and their inclusion within the LANG network boundaries implies some level of functional heterogeneity within the LANG network.

Notably, regions inside LANG that were active for AUDLOC were found closest to auditory cortex. This organization, of a thin sliver of regions encircling primary auditory cortex and juxtaposed between auditory cortex and the LANG network, was previously described using a functional connectivity approach (see intermediate network in the seventh and eighth figures ofBraga et al., 2020). This organization also resembles the organization of speech- or articulation-sensitive regions described byNorman-Haignere et al. (2015)andde Heer et al. (2017), and may thus support some aspect of speech–sound or phoneme processing, even when the sounds do not contain comprehensible words. We investigated whether increasing the number of clusters used for network definition (to 24, from the 14–15 used in main analyses) would yield clusters which separated these speech- or articulation-sensitive auditory responses from the rest of the LANG network. However, this analysis led to inconsistent networks across the group.

## 4 Discussion

### 4.1 Overview

We investigated the properties of a distributed large-scale network that is associated with language processing (LANG). We used intrinsic functional connectivity (iFC) of resting-state data to define LANG as an intrinsically connected network that included classic language regions in inferior frontal (i.e., “Broca’s area”) and lateral temporal cortex (i.e., “Wernicke’s area”;Geschwind, 1965) in eight individuals (Fig. 1). We then showed that the iFC-defined LANG network responds to comprehensible linguistic stimuli presented both through visual (writing;Fig. 2) and auditory (speech;Fig. 3) input, and that the responses closely overlapped the network as estimated using iFC. The LANG network included multiple regions along the superior temporal cortex that were adjacent to auditory cortex, but were clearly dissociated from unimodal auditory functions by an auditory localizer task contrast (AUDLOC, distorted speech clips > fixation,Figs. 4&5). Taken together, these findings advance our understanding of the language network as a transmodal system that works closely with adjacent, but functionally distinct, unimodal sensory areas.

### 4.2 The distributed language network serves a transmodal language function

Previous work has linked the iFC-defined LANG with regions active during reading (Braga et al., 2020), and other work has shown that a similar set of regions activate during both visual and auditory language stimuli (Fedorenko et al., 2010;Scott et al., 2017). Here, we build on these results to show that auditory and visual language tasks both activate overlapping regions that are well encapsulated by the iFC-based estimate of the LANG network (Fig. 3). Thus, our results support that iFC is capable of mapping transmodal language functions within individuals.

This finding has high potential value, as language mapping is routinely performed for clinical purposes. For instance, mapping of language functions in individuals with language difficulties (i.e., aphasias) could inform prognoses by characterizing the extent of damage to language regions (Heiss & Kidwell, 2014;Saur et al., 2010;Schneck et al., 2021). Similarly, for patients with epilepsy or brain tumors, language is routinely used for surgical planning of resections (Tharin & Golby, 2007), guiding which areas may be removed while sparing language ability. A major limitation has been that some patients, particularly those with aphasia (but also those with other forms of dementia), may not be able to perform language tasks. Relatedly, task engagement may be hindered by scanning conditions (e.g., scanner noise during an auditory task, eyesight problems during a reading task), difficulties with task instructions, or participants failing to maintain attention to stimuli. These complications can prohibit conventional task-based language tasks from accurately activating language regions. Our current results indicate that iFC may provide a relatively accurate delineation of unimodal and transmodal language functions using only resting-state data, circumventing many of these potential obstacles.

### 4.3 The distributed language network demarcates transmodal and auditory functions

Both language localizer tasks used here targeted sentence comprehension, which encompasses a broad range of linguistic (e.g., lexical, combinatorial) functions. Both tasks also attempted to limit activation of unimodal sensory cortices (visual areas in READLOC, auditory areas in SPEECHLOC) as well as activity related to general task performance (e.g., task timing, attending to a screen, pressing buttons) through control conditions that employed similar stimuli and demands (Fedorenko et al., 2010;Scott et al., 2017). We were also able to assess the separability of transmodal from unimodal processing by focusing on the AUDLOC contrast. This map, which represented the contrast of listening to incomprehensible speech sounds against a passive fixation baseline, revealed robust bilateral activation within or near auditory cortex (Fig. 4, right;Dick et al., 2017;Formisano et al., 2003;Humphries et al, 2010;Rauschecker & Scott, 2009), supporting that the AUDLOC contrast was an effective activator of auditory areas. These activations also aligned with the iFC-defined AUD network (Fig. 4; left). Thus, despite being defined using independent task data (REST vs. SPEECHLOC) and different approaches (iFC vs. task activation), the boundaries of the LANG network nicely demarcated regions likely serving auditory functions (i.e., sounds that did not contain comprehensible words), and separated them from regions serving transmodal language functions (i.e., active for both SPEECHLOC and READLOC).

Further support for this demarcation was found by considering the overlap between the AUDLOC and READLOC task contrast maps (Fig. 5). We found that the boundaries of the LANG network encapsulated regions active during reading (Braga et al., 2020), and that generally these reading-related regions were not activated by the distorted speech clips. This was informative because the maps were defined in independent data: the distorted speech condition was not used as a control for the reading task, and, therefore, their lack of overlap was not dictated by the analysis strategy. Notably, the AUDLOC contrast map overlapped with regions that were activated by the SPEECHLOC contrast but which did not overlap with the READLOC contrast (and were outside of the LANG network). This separation suggests that the SPEECHLOC map included both auditory regions and transmodal regions (despite the subtraction of the control condition) – a distinction that was well-captured by the iFC maps and the comparison of READLOC and AUDLOC maps (Fig. 5). The overarching findings bolster the claim that the LANG network serves a transmodal language function, despite being closely juxtaposed with regions supporting unimodal auditory functions.

We previously noted that the transmodal language network region in the posterior middle frontal gyrus appears adjacent to orofacial motor regions that are activated by tongue movements (Braga et al., 2020). These regions are functionally linked to one another, and their proximity might facilitate the sharing of information during speech production. Here, we show that a similar pattern is evident in lateral temporal cortex between the transmodal language network and auditory regions.

The close juxtaposition of transmodal language regions and auditory regions in the lateral temporal lobe extends the proposal byKrubitzer (2007)that the location of Broca’s area in inferior frontal cortex is a consequence of the proximity of premotor and motor regions controlling the orofacial musculature. Similarly, transmodal language areas may have evolved in the lateral temporal cortex due to the proximity of auditory areas that process speech sounds. This could explain why the LANG network includes extensive lateral temporal regions that encircle auditory cortex. Our findings contribute to this interpretation by showing that these regions are closely positioned, potentially sharing a boundary, when mapping is performed within the individual brain.

We have here referred to LANG as a transmodal language network, but note that other language-related functions occur outside of these regions. Our interpretation is consistent with the model described byFedorenko et al. (2024), in which regions serving “core” language functions (e.g., combinatorial, lexical) communicate with unimodal networks to integrate motor and perceptual aspects of language. Further, evidence supports that the representation of the meaning of words and phrases is much more broadly encoded (Binder et al., 2009;Huth et al., 2016), such that the “core” language network likely does not encode all semantic representations, even if it activates preferentially for meaningful words and phrases. Therefore, we interpret the transmodal language network here as one which serves these “core” language functions, but does so through interactions with unimodal sensorimotor systems as well as other transmodal areas that encode semantic representations (Fedorenko et al., 2024).

### 4.4 Incomprehensible speech activates the boundary between LANG and AUD

Although the auditory localizer stimuli were successful in activating auditory cortex, the distorted speech stimuli still included recognizable aspects of speech. The filtering procedure muffled words to make comprehension difficult while preserving the intonation and prosody of each speaker (followingScott et al., 2017). Thus, the AUDLOC contrast likely still preserved activation of speech-specialized regions, including those supporting suprasegmental features of speech (e.g. prosody, intonation, rate of speech). Such features may explain why the auditory localizer activity often extended into the language network. Importantly, this interpretation remains consistent with the idea that the LANG regions encode a transmodal, not purely auditory, function. These results imply that there may be additional heterogeneity within the LANG network, as mapped with iFC, that might support intonation- or prosody-level speech processing. Interestingly, the overlap with activity during reading (READLOC;Fig. 5) implies that these processes are recruited by either visual or auditory language stimuli, suggesting they are also transmodal to some degree. To confirm this supposition, future studies might use fully non-linguistic acoustic stimuli (e.g., synthesized tones, music) and higher resolution data to yield further insights into the separability of these functions.

Notably, in addition to the regions that encircled auditory cortex, in most individuals, the AUDLOC maps included frontal lobe regions, close to the ventral motor strip, that likely do not serve auditory processing. Thus the AUDLOC contrast may have also activated non-auditory regions, further supporting the hypothesis that some of the AUDLOC active regions were also transmodal. Prior work has shown that a similar distribution of regions, encircling auditory cortex in the temporal lobe and including regions near precentral cortex, may serve articulatory or phonetic processes (Chang et al., 2010;de Heer et al., 2017;Leonard et al., 2024;Norman-Haignere et al., 2015). These findings imply that the processing of phonemic features may occur through coordination of activity within a network that links speech-sensitive auditory areas with prefrontal regions surrounding the orofacial motor strip. We previously noted that this articulatory/phonemic network may be situated exactly in between transmodal language areas and both auditory and motor cortices (see the “INT” network inBraga et al., 2020).

### 4.5 Limitations

While all participants completed the same number of runs for each task, we excluded some runs because of head motion or inconsistent behavioral responses. As a result, task activity maps were built from different quantities of data depending on the participant. For READLOC, four participants had all eight runs included, two participants had seven runs, one participant (S7) had five runs, and one participant (S4) had four runs (~19 minutes). This last participant predictably showed less robust language activity for this task compared with the other participants. Nevertheless, the study that originated this visual language task reported that ~15–20 minutes was sufficient to identify the language network (Fedorenko et al., 2010). Performance was more consistent for SPEECHLOC, with five participants including all eight runs, and the remaining three participants including seven runs. Participant S4 demonstrated a more robust activity map for this task. Thus, additional data may have improved consistency of results across participants.

A further limitation is that neither task required participants to demonstrate language comprehension. In theory, both tasks could be performed by ignoring the stimuli and simply waiting to respond to the button-press cue. If this were the case, however, we might expect activations to be comparable during both the comprehensible and incomprehensible conditions, rather than revealing classic language regions (and overlapping with the LANG network). Prior work has shown that the passive version of the language localizer task used here (Fedorenko et al., 2010;Scott et al., 2017) yields very similar activation to a version which requires a button press for recognized words (Diachek et al., 2020;Fedorenko et al., 2010). Further work could assess the extent to which these factors influence the degree of overlap between task activation and the iFC-defined network.

Although we labeled regions active for both SPEECHLOC and READLOC tasks as “transmodal” and non-overlapping regions as “unimodal,” it is possible that differences in activation between the two tasks could be the result of factors besides sensory input modality. We observed broader activation for SPEECHLOC compared with READLOC, perhaps as a result of more engaging stimuli (story excerpts vs. individual sentences) or a less tightly controlled contrast condition in the SPEECHLOC task. Thus, while our results suggest that the differences between tasks do reflect differences in input modality to some degree (e.g., we observed activation of auditory areas as one key difference between SPEECHLOC and READLOC), future work could explore what other factors affect activation differences between these tasks.

Although our results show that functional connectivity separates juxtaposed auditory and transmodal language regions, we also observed regions of overlap where auditory and transmodal regions shared a boundary. This overlap could be a consequence of threshold choice, or the fact that the data are inherently blurry (e.g. due to local autocorrelation or spatial smoothing during pre-processing), or could be taken to suggest that responses gradually change from auditory to higher-order language sensitive as one moves along the lateral temporal cortex. Another possible option is that there are specific regions of the auditory hierarchies that are specialized for processing features of speech sounds (e.g. intonation and prosody), which are positioned exactly in between the language and auditory networks (Braga et al., 2020;Norman-Haignere et al., 2015). Future studies might use fully non-linguistic acoustic stimuli (e.g., synthesized tones, music) and higher resolution fMRI data to yield further insights into the organization of these functions.

Finally, here we focused on the lateral temporal cortex and the interface between auditory and transmodal aspects of language. Future work could also assess whether the same principles extend to the interface between visual and transmodal aspects of language in reading.

## 5 Conclusions

We show that the iFC-defined distributed language network is recruited during processing of language from multiple input modalities, and, therefore, supports a transmodal language function. In addition, we demonstrate that the boundaries of this network when defined within individual participants are a relatively good localizer for the separation between unimodal auditory and transmodal language functions. Further work is needed to determine the exact nature of this separation, including how factors such as intonation and prosody affect the underlying regions. Our results suggest that iFC performed within individuals is a useful tool for understanding the complex interactions of cognitive functions underlying language abilities.

## Supplementary Material

Supplementary Material

## Data Availability

All data needed to evaluate the conclusions in the paper are present in the paper or on Dryad (https://doi.org/10.5061/dryad.ngf1vhj4t).

## References

[IMAG.a.25-b1] Alexander-Bloch , A. F. , Shou , H. , Liu , S. , Satterthwaite , T. D. , Glahn , D. C. , Shinohara , R. T. , Vandekar , S. N. , & Raznahan , A. ( 2018 ). On testing for spatial correspondence between maps of human brain structure and function . Neuroimage , 178 , 540 – 551 . 10.1016/j.neuroimage.2018.05.070 29860082 PMC6095687

[IMAG.a.25-b58] Binder , J. R. , Desai , R. H. , Graves , W. W. , & Conant , L. L. ( 2009 ). Where is the semantic system? A critical review and meta-analysis of 120 functional neuroimaging studies . Cerebral Cortex , 19 ( 12 ), 2767 – 2796 . 10.1093/cercor/bhp055 19329570 PMC2774390

[IMAG.a.25-b2] Binder , J. R. , Frost , J. A. , Hammeke , T. A. , Cox , R. W. , Rao , S. M. , & Prieto , T. ( 1997 ). Human brain language areas identified by functional magnetic resonance imaging . Journal of Neuroscience , 17 ( 1 ), 353 – 362 . 10.1523/JNEUROSCI.17-01-00353.1997 8987760 PMC6793702

[IMAG.a.25-b3] Biswal , B. , Zerrin Yetkin , F. , Haughton , V. M. , & Hyde , J. S. ( 1995 ). Functional connectivity in the motor cortex of resting human brain using echo‐planar MRI . Magnetic Resonance in Medicine , 34 ( 4 ), 537 – 541 . 10.1002/mrm.1910340409 8524021

[IMAG.a.25-b4] Braga , R. M. , & Buckner , R. L. ( 2017 ). Parallel interdigitated distributed networks within the individual estimated by intrinsic functional connectivity . Neuron , 95 ( 2 ), 457 – 471 . 10.1016/j.neuron.2017.06.038 28728026 PMC5519493

[IMAG.a.25-b5] Braga , R. M. , DiNicola , L. M. , Becker , H. C. , & Buckner , R. L. ( 2020 ). Situating the left-lateralized language network in the broader organization of multiple specialized large-scale distributed networks . Journal of Neurophysiology , 124 ( 5 ), 1415 – 1448 . 10.1152/jn.00753.2019 32965153 PMC8356783

[IMAG.a.25-b6] Braga , R. M. , Van Dijk , K. R. , Polimeni , J. R. , Eldaief , M. C. , & Buckner , R. L. ( 2019 ). Parallel distributed networks resolved at high resolution reveal close juxtaposition of distinct regions . Journal of Neurophysiology , 121 ( 4 ), 1513 – 1534 . 10.1152/jn.00808.2018 30785825 PMC6485740

[IMAG.a.25-b7] Branco , P. , Seixas , D. , & Castro , S. L. ( 2020 ). Mapping language with resting‐state functional magnetic resonance imaging: A study on the functional profile of the language network . Human Brain Mapping , 41 ( 2 ), 545 – 560 . 10.1002/hbm.24821 31609045 PMC7268076

[IMAG.a.25-b8] Chang , E. F. , Rieger , J. W. , Johnson , K. , Berger , M. S. , Barbaro , N. M. , & Knight , R. T. ( 2010 ). Categorical speech representation in human superior temporal gyrus . Nature neuroscience , 13 ( 11 ), 1428 – 1432 . 10.1038/nn.2641 20890293 PMC2967728

[IMAG.a.25-b9] Cox , R. W. ( 1996 ). AFNI: Software for analysis and visualization of functional magnetic resonance neuroimages . Computers and Biomedical Research , 29 ( 3 ), 162 – 173 . 10.1006/cbmr.1996.0014 8812068

[IMAG.a.25-b10] Cox , R. W. ( 2012 ). AFNI: What a long strange trip it’s been . Neuroimage , 62 ( 2 ), 743 – 747 . 10.1016/j.neuroimage.2011.08.056 21889996 PMC3246532

[IMAG.a.25-b11] Diachek , E. , Blank , I. , Siegelman , M. , Affourtit , J. , & Fedorenko , E. ( 2020 ). The domain-general multiple demand (MD) network does not support core aspects of language comprehension: A large-scale fMRI investigation . Journal of Neuroscience , 40 ( 23 ), 4536 – 4550 . 10.1523/JNEUROSCI.2036-19.2020 32317387 PMC7275862

[IMAG.a.25-b12] Dick , F. K. , Lehet , M. I. , Callaghan , M. F. , Keller , T. A. , Sereno , M. I. , & Holt , L. L. ( 2017 ). Extensive tonotopic mapping across auditory cortex is recapitulated by spectrally directed attention and systematically related to cortical myeloarchitecture . Journal of Neuroscience , 37 ( 50 ), 12187 – 12201 . 10.1523/JNEUROSCI.1436-17.2017 29109238 PMC5729191

[IMAG.a.25-b13] Edmonds , D. , Salvo , J. J. , Anderson , N. , Lakshman , M. , Yang , Q. , Kay , K. ,… & Braga , R. M. ( 2024 ). The human social cognitive network contains multiple regions within the amygdala . Science Advances , 10 ( 47 ), eadp0453 . 10.1126/sciadv.adp0453 39576857 PMC11584017

[IMAG.a.25-b14] Fedorenko , E. , Hsieh , P. J. , Nieto-Castañón , A. , Whitfield-Gabrieli , S. , & Kanwisher , N. ( 2010 ). New method for fMRI investigations of language: Defining ROIs functionally in individual subjects . Journal of Neurophysiology , 104 ( 2 ), 1177 – 1194 . 10.1152/jn.00032.2010 20410363 PMC2934923

[IMAG.a.25-b15] Fedorenko , E. , Ivanova , A. A. , & Regev , T. I. ( 2024 ). The language network as a natural kind within the broader landscape of the human brain . Nature Reviews Neuroscience , 25 ( 5 ), 289 – 312 . 10.1038/s41583-024-00802-4 38609551 PMC13222024

[IMAG.a.25-b16] Fedorenko , E. , & Thompson-Schill , S. L. ( 2014 ). Reworking the language network . Trends in Cognitive Sciences , 18 ( 3 ), 120 – 126 . 10.1016/j.tics.2013.12.006 24440115 PMC4091770

[IMAG.a.25-b17] Felleman , D. J. , & Van Essen , D. C. ( 1991 ). Distributed hierarchical processing in the primate cerebral cortex . Cerebral Cortex , 1 ( 1 ), 1 – 47 . 10.1093/cercor/1.1.1-a 1822724

[IMAG.a.25-b18] Fischl , B. , Sereno , M. I. , & Dale , A. M. ( 1999 ). Cortical surface-based analysis: II: inflation, flattening, and a surface-based coordinate system . Neuroimage , 9 ( 2 ), 195 – 207 . 10.1006/nimg.1998.0396 9931269

[IMAG.a.25-b19] Formisano , E. , Kim , D. S. , Di Salle , F. , Van de Moortele , P. F. , Ugurbil , K. , & Goebel , R. ( 2003 ). Mirror-symmetric tonotopic maps in human primary auditory cortex . Neuron , 40 ( 4 ), 859 – 869 . 10.1016/S0896-6273(03)00669-X 14622588

[IMAG.a.25-b20] Geschwind , N. ( 1965 ). Disconnexion syndromes in animals and man . Brain , 88 ( 3 ), 585 . 10.1093/brain/88.3.585 5318824

[IMAG.a.25-b21] Glasser , M. F. , Coalson , T. S. , Robinson , E. C. , Hacker , C. D. , Harwell , J. , Yacoub , E. , Ugurbil , K. , Andersson , J. , Beckmann , C. F. , Jenkinson , M. , Smith , S. M. , & Van Essen , D. C. ( 2016 ). A multi-modal parcellation of human cerebral cortex . Nature , 536 ( 7615 ), 171 – 178 . 10.1038/nature18933 27437579 PMC4990127

[IMAG.a.25-b22] Goldman-Rakic , P. S. ( 1988 ). Topography of cognition: Parallel distributed networks in primate association cortex . Annual Review of Neuroscience , 11 , 137 – 156 . 10.1146/annurev.ne.11.030188.001033 3284439

[IMAG.a.25-b23] Gordon , E. M. , Laumann , T. O. , Gilmore , A. W. , Newbold , D. J. , Greene , D. J. , Berg , J. J. , Ortega , M. , Hoyt-Drazen , C. , Gratton , C. , Sun , H. , Hampton , J. M. , Coalson , R. S. , Nguyen , A.L. , McDermott , K. B. , Shimony , J. S. , Snyder , A. Z. , Schlaggar , B. L. , Petersen , S. E. , Nelson , S. M. , & Dosenbach , N. U. ( 2017 ). Precision functional mapping of individual human brains . Neuron , 95 ( 4 ), 791 – 807 . 10.1016/j.neuron.2017.07.011 28757305 PMC5576360

[IMAG.a.25-b24] Hacker , C. D. , Laumann , T. O. , Szrama , N. P. , Baldassarre , A. , Snyder , A. Z. , Leuthardt , E. C. , & Corbetta , M. ( 2013 ). Resting state network estimation in individual subjects . Neuroimage , 82 , 616 – 633 . 10.1016/j.neuroimage.2013.05.108 23735260 PMC3909699

[IMAG.a.25-b25] Hagler Jr , D. J. , Hatton , S. , Cornejo , M. D. , Makowski , C. , Fair , D. A. , Dick , A. S. , Sutherland , M. T. , Casey , B. J. , Barch , D. M. , Harms , M. P. , Watts , R. , Bjork , J. M. , Garavan , H. P. , Hilmer , L. , Pung , C. J. , Sicat , C. S. , Kuperman , J. , Bartsch , H. , Xue , F. , … Dale , A. M . ( 2019 ). Image processing and analysis methods for the Adolescent Brain Cognitive Development Study . Neuroimage , 202 , 116091 . 10.1016/j.neuroimage.2019.116091 31415884 PMC6981278

[IMAG.a.25-b26] de Heer , W. A. , Huth , A. G. , Griffiths , T. L. , Gallant , J. L. , & Theunissen , F. E. ( 2017 ). The hierarchical cortical organization of human speech processing . Journal of Neuroscience , 37 ( 27 ), 6539 – 6557 . 10.1523/JNEUROSCI.3267-16.2017 28588065 PMC5511884

[IMAG.a.25-b27] Heiss , W. D. , & Kidwell , C. S. ( 2014 ). Imaging for prediction of functional outcome and assessment of recovery in ischemic stroke . Stroke , 45 ( 4 ), 1195 – 1201 . 10.1161/STROKEAHA.113.003611 24595589 PMC3981064

[IMAG.a.25-b28] Heunis , S. , Breeuwer , M. , Caballero-Gaudes , C. , Hellrung , L. , Huijbers , W. , Jansen , J. F. , Lamerichs , R. , Zinger , S. , & Aldenkamp , A. P. ( 2021 ). The effects of multi-echo fMRI combination and rapid T2*-mapping on offline and real-time BOLD sensitivity . NeuroImage , 238 , 118244 . 10.1016/j.neuroimage.2021.118244 34116148

[IMAG.a.25-b29] Humphries , C. , Liebenthal , E. , & Binder , J. R. ( 2010 ). Tonotopic organization of human auditory cortex . Neuroimage , 50 ( 3 ), 1202 – 1211 . 10.1016/j.neuroimage.2010.01.046 20096790 PMC2830355

[IMAG.a.25-b30] Huth , A. G. , De Heer , W. A. , Griffiths , T. L. , Theunissen , F. E. , & Gallant , J. L. ( 2016 ). Natural speech reveals the semantic maps that tile human cerebral cortex . Nature , 532 ( 7600 ), 453 – 458 . 10.1038/nature17637 27121839 PMC4852309

[IMAG.a.25-b31] Jenkinson , M. , Bannister , P. , Brady , M. , & Smith , S. ( 2002 ). Improved optimization for the robust and accurate linear registration and motion correction of brain images . Neuroimage , 17 ( 2 ), 825 – 841 . 10.1006/nimg.2002.1132 12377157

[IMAG.a.25-b32] Kong , R. , Li , J. , Orban , C. , Sabuncu , M. R. , Liu , H. , Schaefer , A. , Sun , N. , Zuo , X. N. , Holmes , A. J. , Eickhoff , S. B. , & Yeo , B. T. ( 2019 ). Spatial topography of individual-specific cortical networks predicts human cognition, personality, and emotion . Cerebral Cortex , 29 ( 6 ), 2533 – 2551 . 10.1093/cercor/bhy123 29878084 PMC6519695

[IMAG.a.25-b33] Krubitzer , L. ( 2007 ). The magnificent compromise: cortical field evolution in mammals . Neuron , 56 ( 2 ), 201 – 208 . 10.1016/j.neuron.2007.10.002 17964240

[IMAG.a.25-b34] Kwon , Y. H. , Salvo , J. J. , Anderson , N. L. , Edmonds , D. , Holubecki , A. M. , Lakshman , M. , Yoo , K. , Yeo , B. T. T. , Kay , K. , Gratton , C. , & Braga , R. M. ( 2025 ). Situating the salience and parietal memory networks in the context of multiple parallel distributed networks using precision functional mapping . Cell Reports , 44 ( 1 ), 115207 . 10.1016/j.celrep.2024.115207 39826121 PMC11924860

[IMAG.a.25-b35] Laumann , T. O. , Gordon , E. M. , Adeyemo , B. , Snyder , A. Z. , Joo , S. J. , Chen , M. Y. , Gilmore , A. W. , McDermott , K. B. , Nelson , S. M. , Dosenbach , N. U. , Schlaggar , B. L. , Mumford , J. A. , Poldrack , R. A. , & Petersen , S. E. ( 2015 ). Functional system and areal organization of a highly sampled individual human brain . Neuron , 87 ( 3 ), 657 – 670 . 10.1016/j.neuron.2015.06.037 26212711 PMC4642864

[IMAG.a.25-b36] Lee , M. H. , Hacker , C. D. , Snyder , A. Z. , Corbetta , M. , Zhang , D. , Leuthardt , E. C. , & Shimony , J. S. ( 2012 ). Clustering of resting state networks . PLoS One , 7 ( 7 ), e40370 . 10.1371/journal.pone.0040370 22792291 PMC3392237

[IMAG.a.25-b37] Leonard , M. K. , Gwilliams , L. , Sellers , K. K. , Chung , J. E. , Xu , D. , Mischler , G. , Mesgarani , N. , Welkenhuysen , M. , Dutta , B. , & Chang , E. F. ( 2024 ). Large-scale single-neuron speech sound encoding across the depth of human cortex . Nature , 626 ( 7999 ), 593 – 602 . 10.1038/s41586-023-06839-2 38093008 PMC10866713

[IMAG.a.25-b38] Li , J. , Hiersche , K. J. , & Saygin , Z. M. ( 2024 ). Demystifying visual word form area visual and nonvisual response properties with precision fMRI . iScience , 27 ( 12 ), 111481 . 10.1016/j.isci.2024.111481 39759006 PMC11696768

[IMAG.a.25-b39] Lu , J. , Zhao , Z. , Zhang , J. , Wu , B. , Zhu , Y. , Chang , E. F. , Wu , J. , Duffau , H. , & Berger , M. S. ( 2021 ). Functional maps of direct electrical stimulation-induced speech arrest and anomia: A multicentre retrospective study . Brain , 144 ( 8 ), 2541 – 2553 . 10.1093/brain/awab125 33792674 PMC8453410

[IMAG.a.25-b40] Lynch , C. J. , Power , J. D. , Scult , M. A. , Dubin , M. , Gunning , F. M. , & Liston , C. ( 2020 ). Rapid precision functional mapping of individuals using multi-echo fMRI . Cell Reports , 33 ( 12 ), 108540 . 10.1016/j.celrep.2020.108540 33357444 PMC7792478

[IMAG.a.25-b41] Mazziotta , J. C. , Toga , A. W. , Evans , A. , Fox , P. , & Lancaster , J. ( 1995 ). A probabilistic atlas of the human brain: Theory and rationale for its development . Neuroimage , 2 ( 2 ), 89 – 101 . 10.1006/nimg.1995.1012 9343592

[IMAG.a.25-b43] Mesulam , M. M. ( 1998 ). From sensation to cognition . Brain , 121 ( 6 ), 1013 – 1052 . 10.1093/brain/121.6.1013 9648540

[IMAG.a.25-b44] Nobre , A. C. , & McCarthy , G. ( 1995 ). Language-related field potentials in the anterior-medial temporal lobe: II. Effects of word type and semantic priming . Journal of Neuroscience , 15 ( 2 ), 1090 – 1098 . 10.1523/JNEUROSCI.15-02-01090.1995 7869085 PMC6577813

[IMAG.a.25-b45] Norman-Haignere , S. , Kanwisher , N. G. , & McDermott , J. H. ( 2015 ). Distinct cortical pathways for music and speech revealed by hypothesis-free voxel decomposition . Neuron , 88 ( 6 ), 1281 – 1296 . 10.1016/j.neuron.2015.11.035 26687225 PMC4740977

[IMAG.a.25-b46] Poser , B. A. , Versluis , M. J. , Hoogduin , J. M. , & Norris , D. G. ( 2006 ). BOLD contrast sensitivity enhancement and artifact reduction with multiecho EPI: Parallel‐acquired inhomogeneity‐desensitized fMRI . Magnetic Resonance in Medicine , 55 ( 6 ), 1227 – 1235 . 10.1002/mrm.20900 16680688

[IMAG.a.25-b47] Rauschecker , J. P. , & Scott , S. K. ( 2009 ). Maps and streams in the auditory cortex: nonhuman primates illuminate human speech processing . Nature Neuroscience , 12 ( 6 ), 718 – 724 . 10.1038/nn.2331 19471271 PMC2846110

[IMAG.a.25-b48] Saur , D. , Ronneberger , O. , Kümmerer , D. , Mader , I. , Weiller , C. , & Klöppel , S. ( 2010 ). Early functional magnetic resonance imaging activations predict language outcome after stroke . Brain , 133 ( 4 ), 1252 – 1264 . 10.1093/brain/awq021 20299389

[IMAG.a.25-b59] Schneck , S. M. , Entrup , J. L. , Duff , M. C. , & Wilson , S. M. ( 2021 ). Unexpected absence of aphasia following left temporal hemorrhage: A case study with functional neuroimaging to characterize the nature of atypical language localization . Neurocase , 27 ( 1 ), 97 – 105 . https://doi.org/10.1080/13554794.2021.1886309 33666124 10.1080/13554794.2021.1886309 PMC8026574

[IMAG.a.25-b49] Scott , T. L. , Gallée , J. , & Fedorenko , E. ( 2017 ). A new fun and robust version of an fMRI localizer for the frontotemporal language system . Cognitive Neuroscience , 8 ( 3 ), 167 – 176 . 10.1080/17588928.2016.1201466 27386919

[IMAG.a.25-b50] Tharin , S. , & Golby , A. ( 2007 ). Functional brain mapping and its applications to neurosurgery . Operative Neurosurgery , 60 ( 4 ), 185 – 202 . 10.1227/01.NEU.0000255386.95464.52 17415154

[IMAG.a.25-b51] Thomas , T. M. , Singh , A. , Bullock , L. P. , Liang , D. , Morse , C. W. , Scherschligt , X. , Seymour , J. P. , & Tandon , N. ( 2023 ). Decoding articulatory and phonetic components of naturalistic continuous speech from the distributed language network . Journal of Neural Engineering , 20 ( 4 ), 046030 . 10.1088/1741-2552/ace9fb 37487487

[IMAG.a.25-b52] Tisdall , M. D. , Hess , A. T. , Reuter , M. , Meintjes , E. M. , Fischl , B. , & van der Kouwe , A. J. ( 2012 ). Volumetric navigators for prospective motion correction and selective reacquisition in neuroanatomical MRI . Magnetic Resonance in Medicine , 68 ( 2 ), 389 – 399 . 10.1002/mrm.23228 22213578 PMC3320676

[IMAG.a.25-b53] Tomasi , D. , & Volkow , N. D. ( 2012 ). Resting functional connectivity of language networks: Characterization and reproducibility . Molecular Psychiatry , 17 ( 8 ), 841 – 854 . 10.1038/mp.2011.177 22212597 PMC3323720

[IMAG.a.25-b54] Tremblay , P. , & Dick , A. S. ( 2016 ). Broca and Wernicke are dead, or moving past the classic model of language neurobiology . Brain and Language , 162 , 60 – 71 . 10.1016/j.bandl.2016.08.004 27584714

[IMAG.a.25-b55] Turken , A. U. , & Dronkers , N. F. ( 2011 ). The neural architecture of the language comprehension network: Converging evidence from lesion and connectivity analyses . Frontiers in System Neuroscience , 5 , 1 . 10.3389/fnsys.2011.00001 PMC3039157 21347218

[IMAG.a.25-b56] Woolrich , M. W. , Ripley , B. D. , Brady , M. , & Smith , S. M. ( 2001 ). Temporal autocorrelation in univariate linear modeling of FMRI data . Neuroimage , 14 ( 6 ), 1370 – 1386 . 10.1006/nimg.2001.0931 11707093

[IMAG.a.25-b57] Yuan , B. , Xie , H. , Wang , Z. , Xu , Y. , Zhang , H. , Liu , J. , Chen , L. , Li , C. , Tan , S. , Lin , Z. , Hu , X. , Gu , T. , Lu , J. , Liu , D. , & Wu , J. ( 2023 ). The domain-separation language network dynamics in resting state support its flexible functional segregation and integration during language and speech processing . NeuroImage , 274 , 120132 . 10.1016/j.neuroimage.2023.120132 37105337

