# Supplementary material for: Intrinsic functional connectivity delineates transmodal language functions
Source: Imaging Neurosci (Camb). 2025 Jun 10;3:IMAG.a.25. doi: 10.1162/IMAG.a.25 (PMC12319807; doi:10.1162/IMAG.a.25)
Supplement: Supplementary Material [file imag.a.25_supp.pdf]

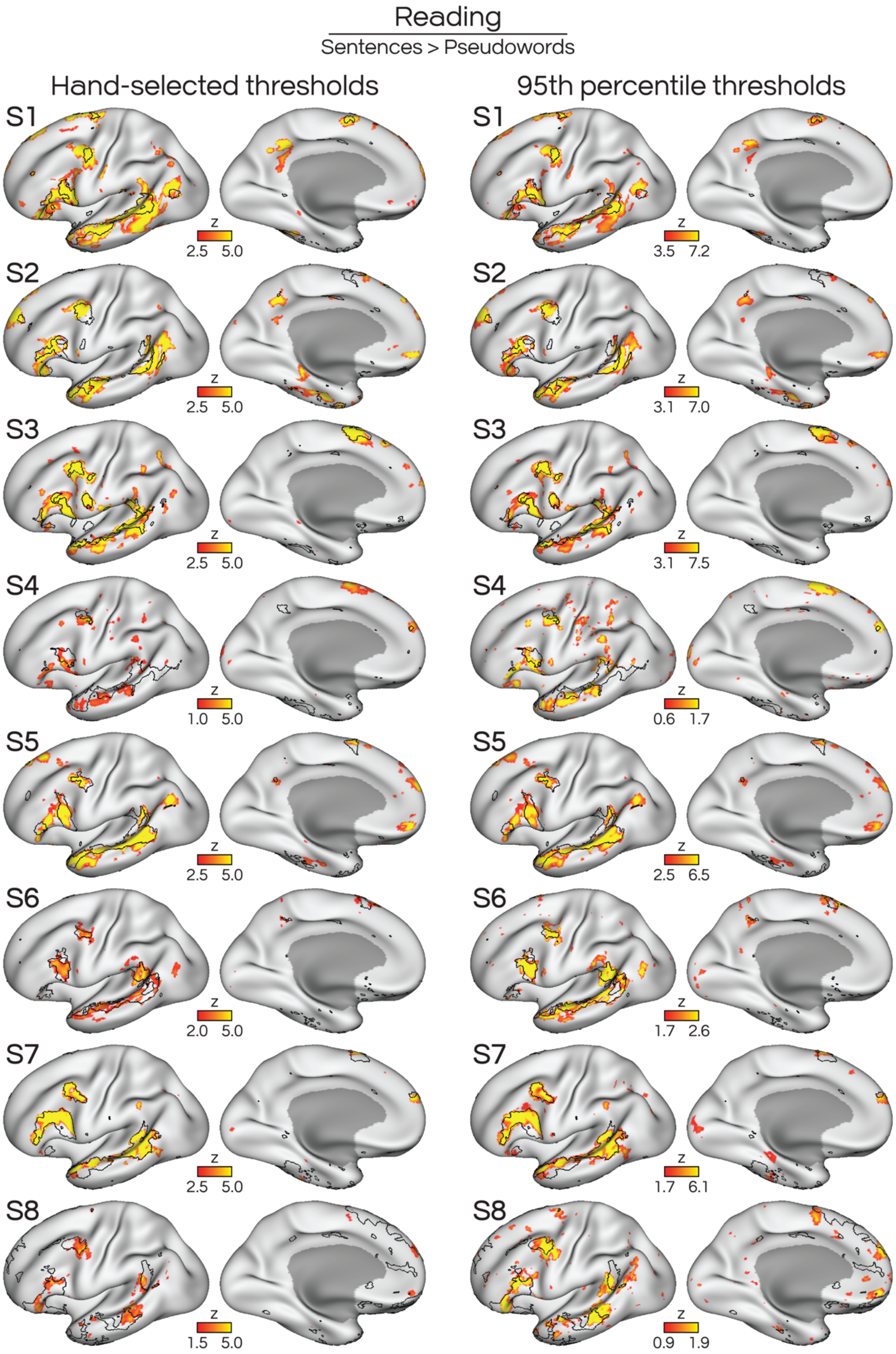

Supplementary Fig. S1: READLOC activations are comparable between qualitative (left) and quantitative (right) thresholds. Task activity is shown using a heatmap color palette, and network boundaries are shown as black borders. Left) Display thresholds were chosen qualitatively per participant, to minimize noise-related "speckling", and to account for signal quality variations between different individuals. Right) Thresholds were also chosen quantitatively by only including vertices with the top 5% intensity values. The maps look similar using both methods.

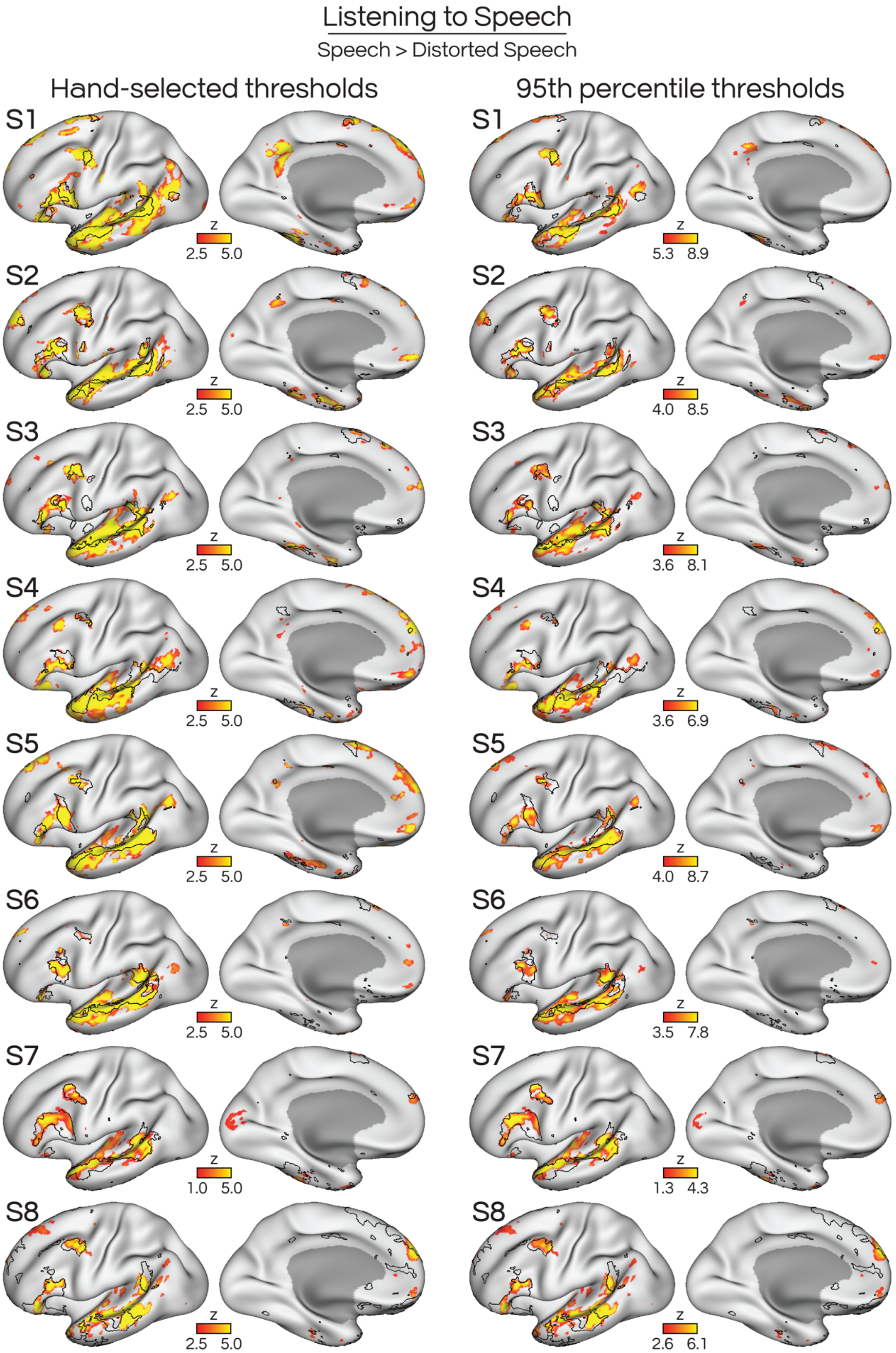

Supplementary Fig. S2: SPEECHLOC activations are comparable between qualitative (left) and quantitative (right) thresholds. Figure formatted according to Supp. Fig. S1.

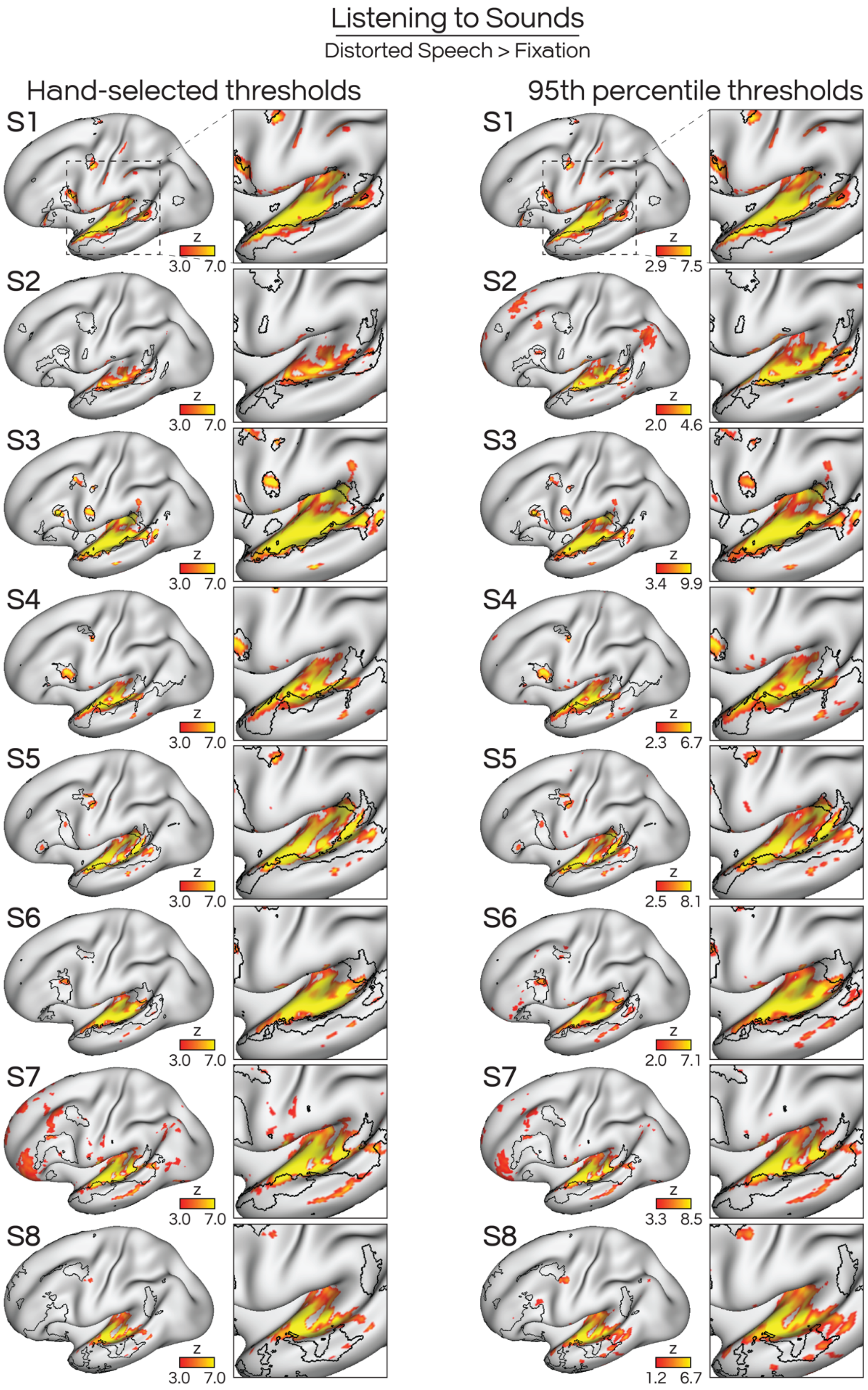

Supplementary Fig. S3: AUDLOC activations are comparable between qualitative (left) and quantitative (right) thresholds. Figure formatted according to Supp. Fig. S1.

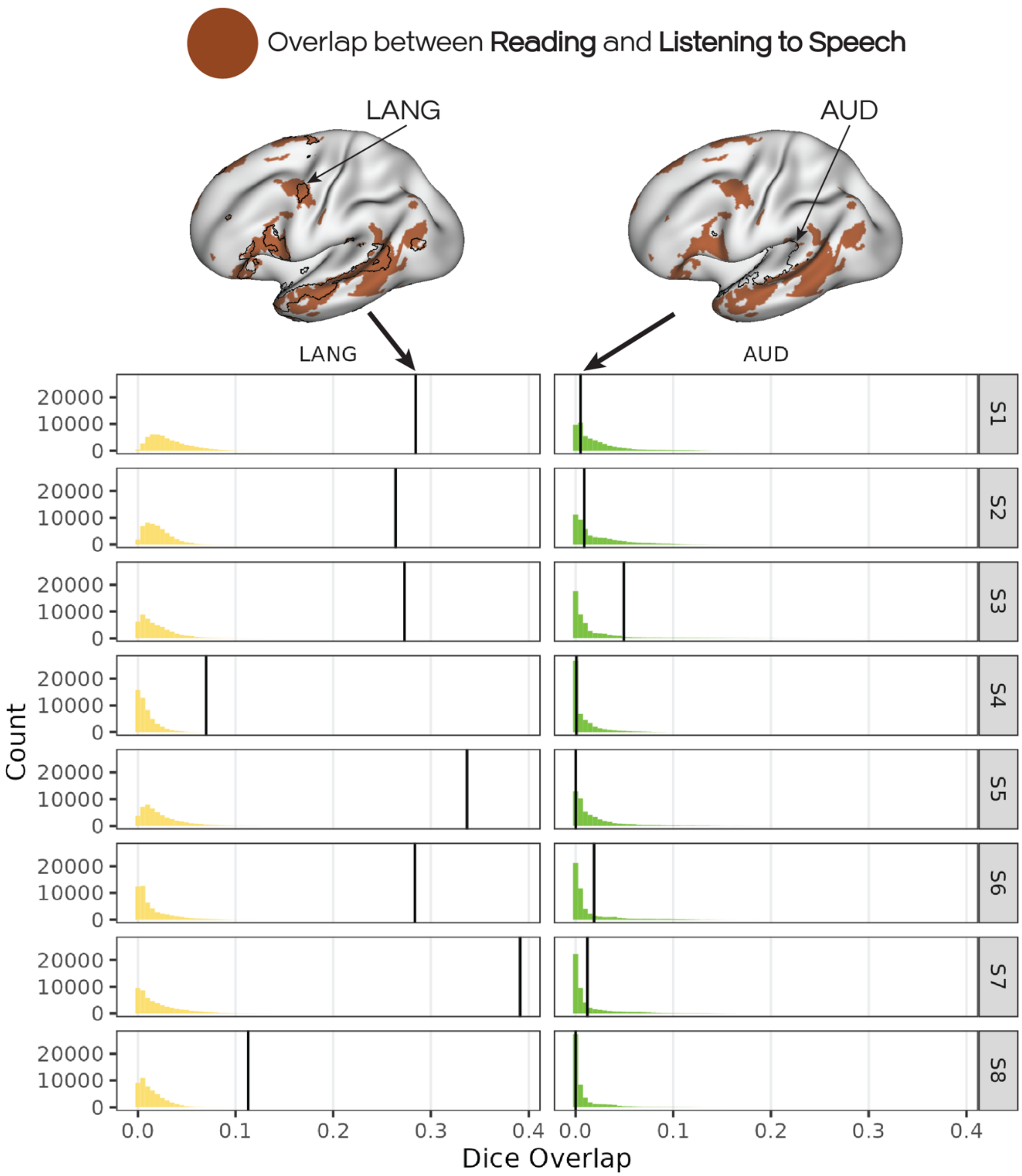

Supplementary Fig. S4: The overlap between the language network (LANG) and regions active for both language contrasts (READLOC and SPEECHLOC) is higher for original orientation compared to random rotations of LANG. For each participant, LANG and auditory network (AUD) regions were each spun around the cortical surface, using the same set of 50,000 randomized rotations. Overlap was quantified for each iteration using Dice coefficients. For LANG, the original orientation of the regions showed greater alignment with transmodal language activity compared to the majority of other rotations (range: 49,943-50,000). For AUD, the original orientation showed average alignment with language activity compared to other rotations. Vertical black bars indicate each participant's Dice coefficient of overlap for the original orientations of LANG and AUD, respectively.

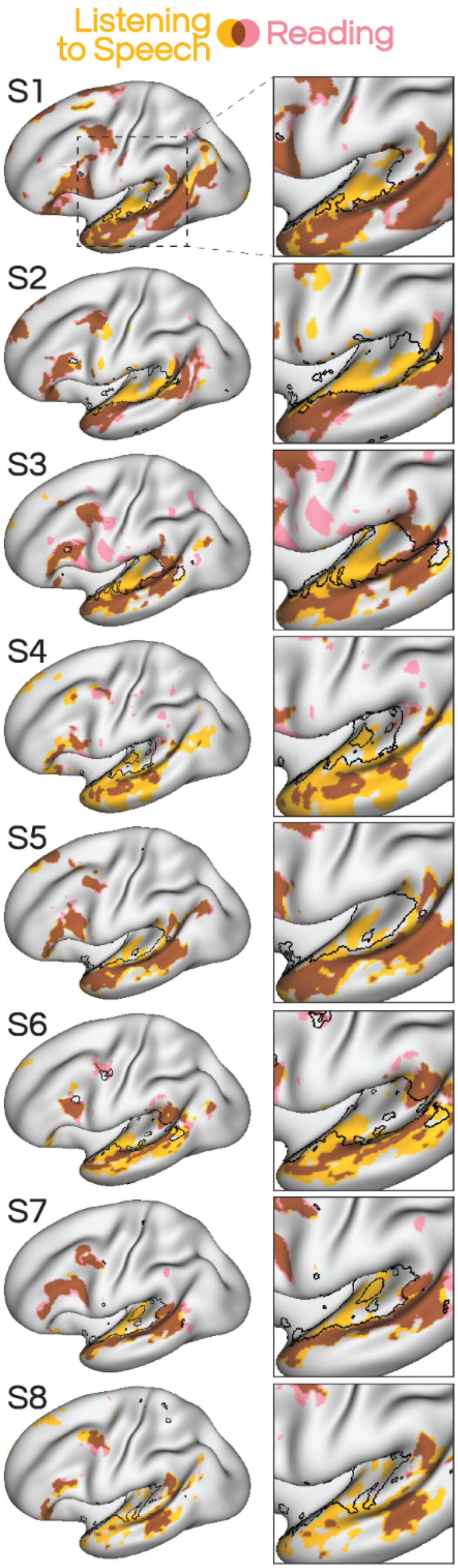

Supplementary Fig. S5: Overlap between READLOC (Reading) and SPEECHLOC (Listening to Speech) contrasts, shown with border of AUD network. The activity maps are identical to main Fig. 3, but here include the border of the AUD network instead of LANG. The AUD network overlaps with a portion of SPEECHLOC activity (yellow, brown), but does not extensively include READLOC activity (red, brown). In many cases, the border of the resting-state functional-connectivity-defined AUD network closely matches the boundaries of task active regions (e.g., see S2, S7). Qualitative display thresholds were used (see Supp. Figs. S1 & S2)

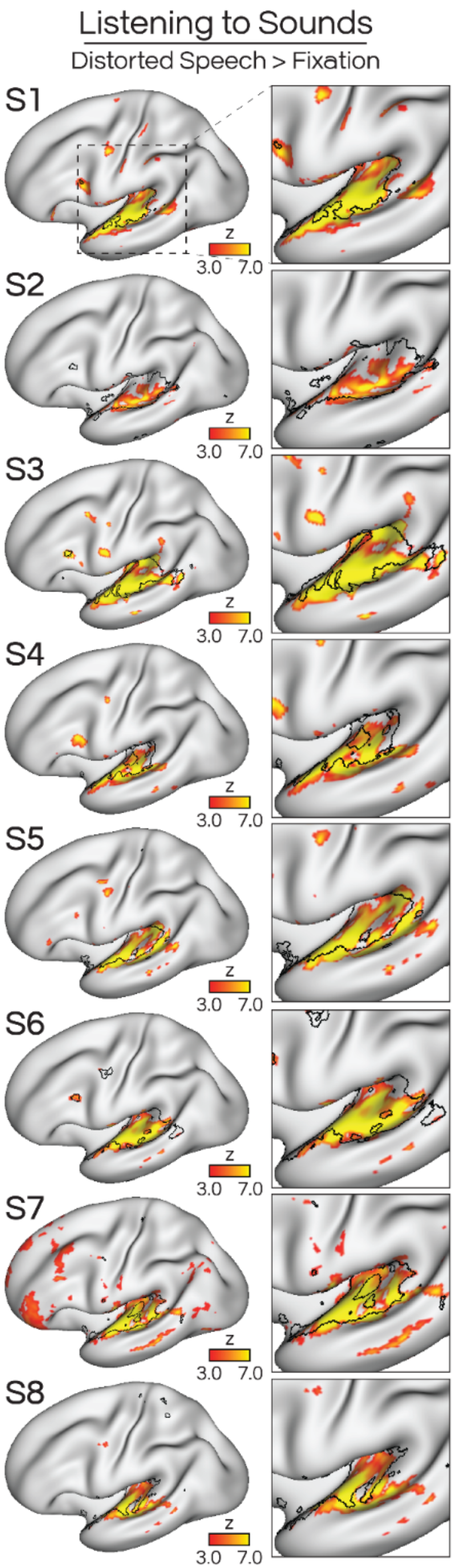

Supplementary Fig. S6: Activity for AUDLOC (Listening to Sounds), shown with border of AUD network. The activity maps are identical to main Fig. 4, but here feature the border of the AUD network instead of LANG. The boundaries of the resting-state functional-connectivity-defined AUD network in many cases encapsulate activity related to listening to sounds.

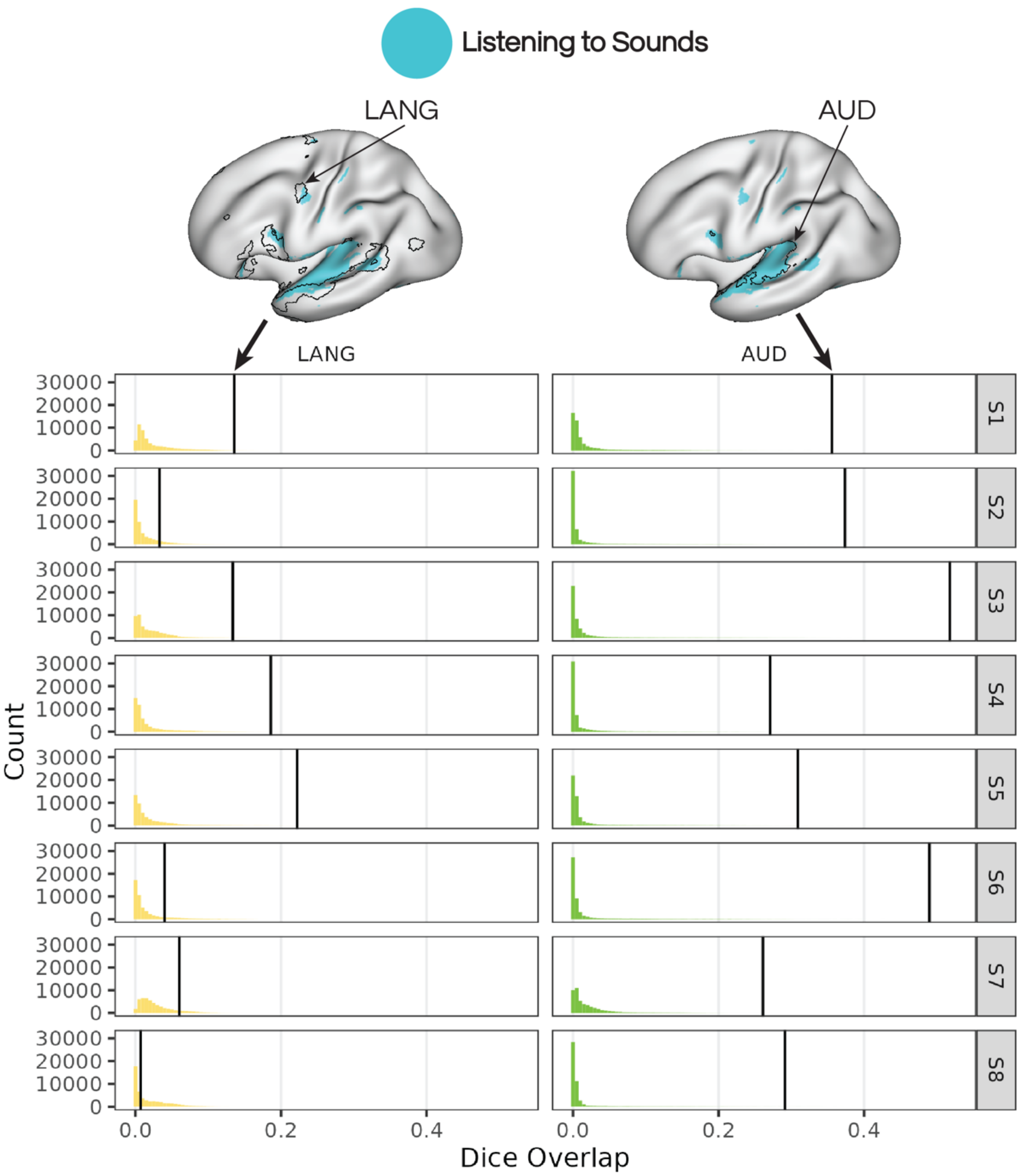

Supplementary Fig. S7: The overlap between the auditory network (AUD) and regions active for the auditory contrast (AUDLOC; Listening to Sounds) is higher for original orientation compared to random rotations of AUD and LANG. For each participant, LANG and AUD regions were spun around the cortical surface, using the same set of 50,000 randomized rotations. Overlap was quantified for each iteration using Dice coefficients. For both LANG and AUD, the original orientation of the regions showed greater alignment with task activity compared to the majority of other rotations. However, this alignment was relatively tighter for AUD compared to LANG (AUD range: 49,807-50,000; LANG range: 23,789-49,899). Vertical black bars indicate each participant's Dice coefficient of overlap for the original orientations of LANG and AUD, respectively.

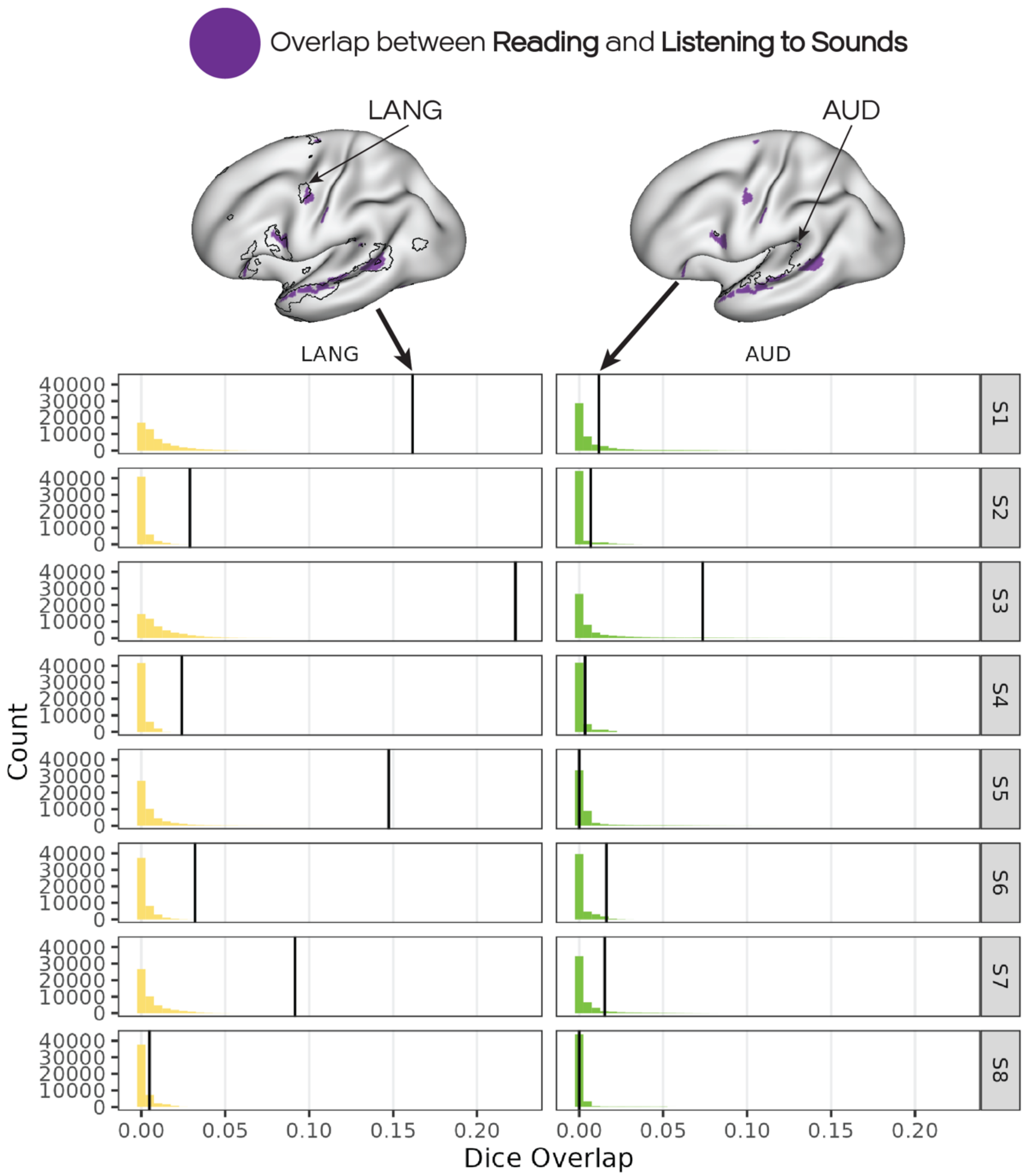

Supplementary Fig. S8: The overlap of the language network (LANG) and regions active for both the reading and listening to sounds contrasts (READLOC and AUDLOC) is higher for original orientation compared to alternate rotations of LANG. For each participant, LANG and auditory network (AUD) regions were each spun around the cortical surface, using the same set of 50,000 randomized rotations. Overlap was quantified for each iteration using Dice coefficients. For LANG, the original orientation of the regions showed greater alignment with regions active for both READLOC and AUDLOC compared to the majority of other rotations (range: 42,769-50,000). For AUD, the original orientation showed average alignment with the active regions compared to other rotations. Vertical black bars indicate each participant's Dice coefficient of overlap for the original orientations of LANG and AUD, respectively.
